# From Pathway Tracing to Actionable Targets: Integrative Mendelian Randomization and Experimental Triangulation Map Metabolic Pathways Across Ovarian Cancer Histotypes

**DOI:** 10.3390/ijms27115043

**Published:** 2026-06-02

**Authors:** Xinqi Wang, Haoyu Wang, Siyuan Hu, Wenyi Zhang, Huiyu Chen, Ying Shen, Hongyang Xue, Li Hong

**Affiliations:** 1 Department of Obstetrics and Gynecology, Pelvic Floor Research Centre of Hubei Province, Renmin Hospital of Wuhan University, No. 238 Jiefang Road, Wuchang District, Wuhan 430060, China; 2 Department of Obstetrics and Gynecology, Peking Union Medical College Hospital, Chinese Academy of Medical Sciences & Peking Union Medical College, Beijing 100730, China; 3 National Clinical Research Center for Women’s Health and Obstetric and Gynecologic Diseases, Peking Union Medical College Hospital, Chinese Academy of Medical Sciences & Peking Union Medical College, Beijing 100730, China

**Keywords:** ovarian cancer, histotypes, metabolomics, pathway-based Mendelian randomization, drug-target Mendelian randomization, lactate

## Abstract

Ovarian cancer (OC) comprises multiple histotypes with distinct mechanisms, molecular features, and clinical behavior. We used Mendelian randomization (MR) to map histotype-stratified metabolic pathways and connect them to drug targets, establishing a translatable target–metabolic node–histotype risk chain. We built a multi-stage MR framework using Integrative Epidemiology Unit (IEU) OpenGWAS summary statistics. After screening 1400 plasma metabolites against overall ovarian cancer in UK Biobank and Ovarian Cancer Association Consortium (OCAC) with KEGG enrichment, we traced a prespecified amino acid/energy–nitrogen axis using histotype-stratified univariable MR and pathway-restricted multivariable MR. We then performed cis drug-target MR for *PPARG*, *DPP4*, *ABCC8/KCNJ11*, and *SLC5A2*, integrated triangulation, colocalization, and mediation analyses, and experimentally interrogated the prioritized *PPARG/ABCC8-KCNJ11*–lactate–invasive mucinous ovarian cancer (IMOC) triangle. Screening nominated 55 and 72 metabolites in UK Biobank and OCAC, respectively (IVW *p* < 0.05), highlighting amino-acid nitrogen and central-carbon metabolism. Univariable Mendelian randomization (UVMR) showed marked heterogeneity: alanine increased low-grade serous ovarian cancer (LGSOC) risk, glutamate was protective for endometrioid OC, and lactate-related traits most consistently implicated the low-grade/borderline serous lineage. In multivariable Mendelian randomization (MVMR), tryptophan and lactate levels emerged as independent risk nodes for serous low-grade plus low malignant potential (LG + LMP). Drug-target MR prioritized *PPARG* as protective (OR = 0.18) and *ABCC8/KCNJ11* as risk-increasing (OR = 7.50) for IMOC, with opposite target → lactate effects supporting a directionally symmetric target–lactate–IMOC triangle. Experimental perturbation in mucinous ovarian cancer models produced concordant reciprocal changes in lactate and malignant phenotypes, extending this triangle biologically. This integrative MR framework delineates histotype-specific metabolic drivers and links them to actionable targets, providing a roadmap from genetic prioritization to mechanistic and translational validation.

## 1. Introduction

Ovarian cancer remains one of the most lethal gynecologic malignancies, largely because early disease often presents with nonspecific symptoms, many patients are diagnosed at an advanced stage, and effective strategies for prevention and early detection are still limited [1,2]. According to the Global Cancer Observatory (GLOBOCAN), an estimated 324,603 new cases and 206,956 deaths occurred worldwide in 2022, underscoring its substantial public health burden [3]. Recent epidemiologic reviews further highlight marked geographic and temporal variation in epithelial ovarian cancer incidence and mortality, with declines in many economically developed settings but rising incidence in parts of Asia and eastern Europe [4]. Inherited susceptibility also remains an important component of ovarian cancer epidemiology; recent age-specific data in Asian *BRCA1/2* pathogenic variant carriers further underscore the clinical relevance of hereditary risk stratification [5]. Importantly, ovarian cancer is not a single disease entity. Based on contemporary pathologic and molecular evidence, epithelial ovarian cancer can be broadly categorized into five major histotypes—high-grade serous, low-grade serous, endometrioid, clear cell, and mucinous carcinomas—which differ markedly in etiology, molecular features, and clinical behavior [6]. Therefore, etiologic determinants (including metabolic factors) are likely to be histotype-dependent, motivating systematic causal investigations within a histotype-stratified framework.

Metabolic reprogramming is a hallmark of cancer and provides a mechanistic link between the host’s systemic metabolic state, the tumor microenvironment, and tumor progression [7,8]. Beyond alterations in glucose metabolism, cancer cells remodel broader carbon and nitrogen metabolic networks [8,9]. For example, lactate is no longer viewed solely as a by-product of glycolysis but also as a signaling metabolite shaping the tumor microenvironment and immune landscape [10]; dysregulated nitrogen handling can perturb the arginine–ornithine/urea-cycle module, while downstream polyamine metabolism supports proliferation and contributes to immunosuppressive states [11]; and amino acids such as glutamine have been repeatedly implicated as key substrates and therapeutic vulnerabilities, including in ovarian cancer [12]. From the perspective of biochemical connectivity and pathway interpretability, we organized these signals along a predefined mechanistic axis spanning amino acid-related nodes, TCA-cycle intermediates, glycolytic end-products (including lactate), and the urea-cycle/polyamine module, to enable more coherent pathway-level causal mapping relevant to ovarian carcinogenesis. At the same time, circulating metabolomics has expanded the breadth of population-level signals linked to ovarian cancer, yet conventional association studies remain vulnerable to confounding, reverse causation, and the strong correlation structure among metabolites, often obscuring which nodes are truly causal and thereby complicating mechanistic interpretation and intervention prioritization [13].

Mendelian randomization (MR) leverages genetic variants associated with exposures as instrumental variables to strengthen causal inference under explicit assumptions, partly mitigating confounding and reverse causation [14,15,16,17]. To enhance transparency and interpretability, the STROBE-MR reporting guideline provides a structured framework for the design and reporting of MR studies [18]. With the rapid growth of metabolite GWAS resources, metabolome-wide MR has become feasible for systematically prioritizing metabolites and pathways with potential causal relevance [19]. In addition, genetic proxies located near drug-target genes can approximate target perturbation, linking etiologic signals to potentially actionable mechanisms and informing drug development or repurposing [20].

Against this background, we established a multi-stage, two-sample MR framework to systematically characterize metabolic causal determinants of ovarian cancer from a histotype-stratified perspective and to connect these metabolic signals to potentially actionable drug targets. We first performed a metabolome-wide MR screen of 1400 circulating plasma metabolites. We then conducted pathway-guided follow-up analyses centered on the amino acid/energy–nitrogen metabolism axis, with stratification across major histotypes and low malignant potential outcomes. Next, we used pathway-restricted multivariable MR to estimate conditional direct effects within relevant modules. Finally, we evaluated genetic proxies for key glucose-lowering drug targets and integrated cis colocalization, target–metabolite–outcome triangulation, and mediation analyses; we further experimentally interrogated the MR-prioritized target–lactate–IMOC triangle, particularly the *PPARG* and *ABCC8/KCNJ11* arms, in mucinous ovarian cancer models. By coupling pathway-informed causal inference with target-level and experimental triangulation, our study aims to provide more coherent and interpretable evidence for histotype-specific metabolic mechanisms and to prioritize mechanistically grounded hypotheses for ovarian cancer prevention and intervention.

## 2. Results

### 2.1. Metabolome-Wide MR Screening Implicates Amino-Acid and Central-Carbon Metabolism Pathways

**Figure 1:**
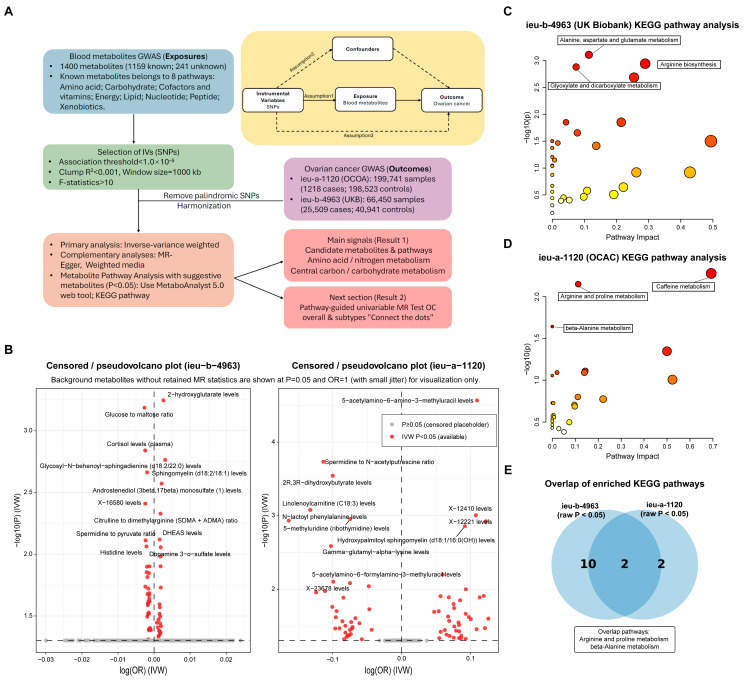
Metabolome-wide Mendelian randomization (MR) screening and pathway enrichment analysis for ovarian cancer (OC). (**A**) Study overview. A total of 1400 circulating blood metabolites in Europeans were analyzed as exposures against two independent overall OC GWAS outcomes (OCAC: OpenGWAS ieu-a-1120; UK Biobank: ieu-b-4963) using two-sample MR (primary method: IVW). (**B**) Censored/placeholder volcano plots. Each panel displays 400 representative metabolites, including all metabolites with retained IVW statistics and IVW *p* < 0.05 plus a random background sample from metabolites without retained MR statistics. Red points indicate metabolites nominated at the prespecified nominal IVW threshold (*p* < 0.05), and gray points indicate placeholder metabolites without retained statistics. Dashed lines mark OR = 1 and *p* = 0.05; labeled points denote the most significant metabolites per outcome. (**C**,**D**) Post-MR KEGG pathway enrichment and topology analysis based on IVW-positive metabolites (*p* < 0.05) for (**C**) ieu-b-4963 and (**D**) ieu-a-1120 (MetaboAnalyst; KEGG). Bubble size/color/position reflects pathway significance and pathway impact. (**E**) Overlap of nominally enriched pathways (raw *p* < 0.05) between the two outcomes. Abbreviations: MR, Mendelian randomization; IVW, inverse-variance weighted; OR, odds ratio; OC, ovarian cancer; OCAC, Ovarian Cancer Association Consortium; KEGG, Kyoto Encyclopedia of Genes and Genomes.

We first conducted a metabolome-wide two-sample Mendelian randomization (MR) screen of 1400 circulating plasma metabolic traits and compared the nomination results across two independent genome-wide association studies (GWAS) of overall ovarian cancer (OC) (UK Biobank: ieu-b-4963; OCAC: ieu-a-1120) (Figure 1A; Table 1; Supplementary Tables S1 and S2) [19,21]. Using nominal IVW *p* < 0.05 as the prespecified nomination threshold, 55 metabolites were nominated in the UK Biobank overall OC and 72 metabolites were nominated in the OCAC overall OC (Figure 1B; Supplementary Table S3). These nominated signals spanned diverse biochemical classes, including amino-acid derivatives/conjugates, nucleoside/nucleotide-related traits, bile-acid derivatives, polyamine-related ratios, and multiple lipid subclasses. Only a small subset of metabolites showed consistent nominal significance across multiple MR methods (Supplementary Figure S1; Supplementary Table S3).

To move beyond isolated “hits” and assess whether the screen converged on coherent biological themes, we performed pathway enrichment analysis using the IVW-positive metabolite sets. In the UK Biobank outcome, KEGG enrichment highlighted nitrogen metabolism and central-carbon metabolism, with alanine/aspartate/glutamate metabolism, arginine biosynthesis, glyoxylate and dicarboxylate metabolism, and arginine and proline metabolism remaining significant after multiple-testing correction (FDR < 0.05) (Figure 1C; Supplementary Table S4). In OCAC, no pathway remained significant after FDR correction, although several pathways were nominally enriched (Figure 1D; Supplementary Table S4) [22,23,24].

Despite limited overlap at the single-metabolite level between the two overall OC outcomes, we observed greater concordance at the pathway level: among nominally significant pathways (raw *p* < 0.05), arginine and proline metabolism and beta-alanine metabolism overlapped across the two outcomes (Figure 1E; Supplementary Table S4). Accordingly, we adopted a pathway-oriented follow-up strategy to place nominated metabolites within explicit mechanistic chains and to guide hypothesis-driven, histotype-stratified follow-up analyses.

### 2.2. Pathway-Tracing Univariable MR Reveals Histotype Heterogeneity Along an Amino-Acid/Energy–Nitrogen Axis

**Figure 2:**
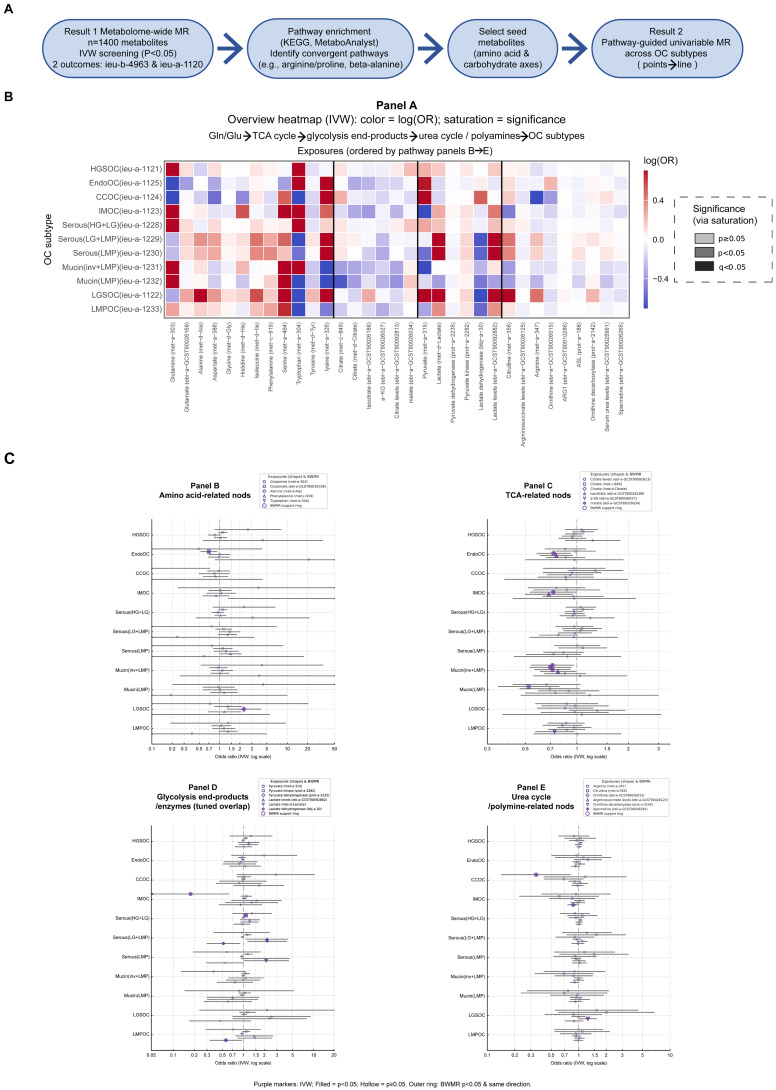
Pathway node-based univariable MR “pathway-tracing” analysis across OC histotypes. (**A**) A predefined “seed-to-pathway” mechanistic axis (amino acid-related nodes → TCA cycle intermediates → glycolysis end products and key enzymes → urea cycle/polyamine-related nodes) was used for stratified univariable MR (UVMR) testing across OC histotypes. (**B**) IVW overview heatmap (Panel A). The *x*-axis denotes key exposure nodes along the mechanistic axis, and the *y*-axis denotes OC outcomes/histotypes (OpenGWAS IDs annotated). Color encodes the direction and magnitude of log(OR), with intensity/saturation indicating significance tiers. (**C**) Module-stratified IVW forest plots (Panels B–E). Points and horizontal lines denote IVW ORs and 95% CIs on a log scale (vertical dashed line, OR = 1). Filled points indicate nominal IVW significance (*p* < 0.05). Outer rings denote BWMR support (same direction and BWMR *p* < 0.05); truncated CIs are shown with arrows. Abbreviations: BWMR, Bayesian weighted MR; TCA, tricarboxylic acid cycle; HGSOC, high-grade serous ovarian cancer; Serous(HG + LG), high-grade and low-grade serous ovarian cancer; Serous(LG + LMP), serous ovarian cancer: low grade and low malignant potential; Serous(LMP), serous ovarian cancer: low malignant potential; LGSOC, low-grade serous ovarian cancer; LMP-OC, low-malignant-potential ovarian cancer; EndoOC, endometrioid ovarian cancer; CCOC, clear cell ovarian cancer; IMOC, invasive mucinous ovarian cancer; Mucin(inv + LMP), mucinous ovarian cancer: invasive and low malignant potential; Mucin(LMP), low-malignant-potential mucinous ovarian cancer.

Building on the metabolome-wide screen, we conducted a hypothesis-driven “pathway-tracing” univariable MR (UVMR) analysis along a predefined nitrogen/energy metabolism axis “amino acids–TCA-cycle intermediates–glycolysis/lactate end-products and key enzymes–urea-cycle/polyamine nodes” (Figure 2A; Table 1). The overview heatmap of Panel A indicated marked heterogeneity across OC histotypes and malignant-potential strata, rather than a single uniform metabolic signature for overall OC (Figure 2B).

Within amino-acid-related nodes (Figure 2C, Panel B), multiple amino acids showed nominal histotype-specific associations. Genetically predicted alanine was nominally associated with increased risk of low-grade serous OC (LGSOC) (OR = 2.28, 95% CI 1.28–4.08; *p* = 0.0053), whereas glutamate was nominally associated with lower risk of endometrioid OC (OR = 0.70, 95% CI 0.52–0.93; *p* = 0.012) (Supplementary Table S5). Phenylalanine showed a nominal risk-increasing association with serous LMP-OC (OR = 1.46, 95% CI 1.08–1.96; *p* = 0.013), and tryptophan showed a nominal risk-increasing association with invasive mucinous OC (IMOC) (OR = 53.85, 95% CI 2.55–1138.67; *p* = 0.010) (Supplementary Table S5). For TCA-cycle intermediates (Figure 2C, Panel C), several metabolites showed nominal protective associations in selected histotypes. Alpha-ketoglutarate was nominally associated with reduced risk of overall LMP-OC (OR = 0.74, 95% CI 0.57–0.96; *p* = 0.025), and citrate was nominally associated with reduced risk of mucinous LMP-OC (OR = 0.52, 95% CI 0.35–0.79; *p* = 0.0021) (Supplementary Table S5). In the glycolysis/pyruvate–lactate module (Figure 2C, Panel D), subtype differentiation was more pronounced. Both lactate and lactate levels were nominally associated with increased risk of serous(LG + LMP) OC (lactate: OR = 2.17, 95% CI 1.14–4.15; *p* = 0.019; lactate levels: OR = 2.16, 95% CI 1.08–4.34; *p* = 0.030), whereas LDH showed a nominal protective association with the same phenotype (OR = 0.52, 95% CI 0.30–0.90; *p* = 0.0189) (Supplementary Table S5). Pyruvate showed a nominal protective association with IMOC (OR = 0.18, 95% CI 0.05–0.70; *p* = 0.013), and pyruvate kinase showed a nominal protective association with serous(LG + LMP) OC (OR = 0.66, 95% CI 0.47–0.93; *p* = 0.016) (Supplementary Table S5). At the urea-cycle/polyamine endpoint (Figure 2C, Panel E), ODC showed a nominal risk-increasing association with LGSOC (OR = 1.26, 95% CI 1.01–1.58; *p* = 0.041), whereas arginine showed a nominal protective association with clear cell OC (OR = 0.34, 95% CI 0.14–0.83; *p* = 0.017) (Supplementary Table S5).

At this stage, Bayesian weighted Mendelian randomization (BWMR) provided directionally concordant support for a subset of the nominal UVMR associations (concordant direction with IVW and BWMR *p* < 0.05; indicated by outer rings in Figure 2C and documented in Supplementary Table S5). Overall, pathway tracing organized the “point signals” from the metabolome-wide screen into a continuous mechanistic chain and underscored substantial histotype heterogeneity. Given the genetic correlation among nodes within each module and the largely nominal nature of many associations, we next applied pathway-restricted multivariable MR to estimate conditional direct effects and to prioritize more plausibly independent driver nodes.

### 2.3. Pathway-Restricted MVMR Prioritizes Independent Conditional Direct Effects Within Metabolic Modules

To estimate conditional direct effects within correlated modules, we performed pathway-restricted MVMR for each metabolic module using union instruments and a common-SNP principle. Selected conditional direct-effect patterns are summarized below, and full model outputs, q values, and diagnostics are provided in Supplementary Table S6 and Supplementary Figures S2–S17.

#### 2.3.1. Aromatic Amino Acids: Tryptophan Emerges as the Clearest FDR-Supported Independent Risk Node

**Figure 3:**
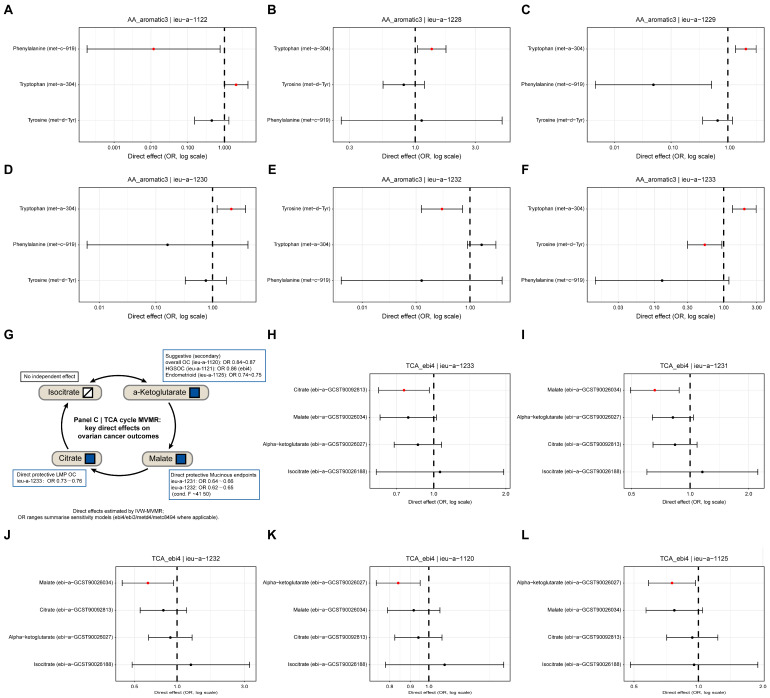
Multivariable MR (MVMR) of aromatic amino acids and TCA-cycle metabolites identifies histotype-specific independent direct effects. (**A**–**F**) IVW-MVMR direct effects (ORs and 95% CIs; log scale) for the AA_aromatic3 model (tryptophan met-a-304, phenylalanine met-c-919, tyrosine met-d-Tyr) across selected OC outcomes/histotypes; red points indicate nominal significance. (**G**) TCA cycle schematic highlighting key metabolites included in MVMR and summarizing the direction/range of direct effects across model specifications; α-ketoglutarate shows lower conditional F in some models. Dashed vertical lines indicate OR = 1, and arrows indicate truncated confidence intervals. (**H**–**L**) Forest plots of IVW-MVMR direct effects for the TCA_ebi4 model in the indicated outcomes. Selected findings are highlighted in the text; full outcome-wide results, q values, and diagnostics are provided in Supplementary Figures S2–S9 and Supplementary Table S6. Abbreviations: MVMR, multivariable MR; IVW, inverse-variance weighted; OR, odds ratio; TCA, tricarboxylic acid cycle.

For aromatic amino acids, we fitted a prespecified aromatic amino-acid MVMR model (AA_aromatic3; tryptophan, phenylalanine, and tyrosine) as the primary specification, with broader/alternative amino-acid models (AminoAcid_main5, AA_transamination4, and AA_onecarbon2) evaluated as sensitivity specifications (Supplementary Figures S2–S5; Supplementary Table S6). In AA_aromatic3 model, only tryptophan retained a stable independent effect after mutual adjustment. Tryptophan was associated with increased risk of serous(LG + LMP) OC (OR = 2.08, 95% CI 1.37–3.16; *p* = 6.11 × 10^–4^; FDR q = 0.0119) (Figure 3C). Tryptophan also showed risk-increasing conditional direct effects for serous LMP-OC (OR = 1.61, 95% CI 1.21–2.14; *p* = 0.0011; q = 0.0127) and overall LMP-OC (OR = 1.99, 95% CI 1.34–2.95; *p* = 6.08 × 10^–4^; q = 0.0119) (Figure 3D,F). By contrast, the direct effects of phenylalanine and tyrosine attenuated or were unstable after adjustment (Figure 3A–F; Supplementary Figures S2–S5; Supplementary Table S6).

#### 2.3.2. TCA-Cycle Intermediates: Malate Shows Suggestive Protective Conditional Direct-Effect Signals for Mucinous Phenotypes

For TCA-cycle intermediates, we implemented a primary four-node TCA model (TCA_ebi4: citrate, isocitrate, α-ketoglutarate, and malate), complemented by a reduced model (TCA_ebi3) and alternative exposure definitions (TCA_metd4 and TCA_metc8494) to assess robustness (Supplementary Figures S6–S9; Supplementary Table S6). In the TCA_ebi4 model (Figure 3G–L), most UVMR signals attenuated after conditioning, consistent with shared genetic architecture within the module. Notably, citrate showed a suggestive protective conditional direct effect for overall LMP-OC (OR = 0.75, 95% CI 0.59–0.96; *p* = 0.0248) (Figure 3H). Malate showed suggestive protective conditional direct effects for mucinous-related phenotypes, including mucinous (invasive + LMP) OC (OR = 0.66, 95% CI 0.49–0.88; *p* = 0.0062) and mucinous LMP-OC (OR = 0.62, 95% CI 0.41–0.95; *p* = 0.030), with conditional F approximately 41 (Figure 3I,J; Supplementary Table S6). However, none of the TCA-module findings remained significant after within-model multiple-testing correction (Supplementary Table S6), and these results should therefore be interpreted as suggestive rather than FDR-supported.

#### 2.3.3. Terminal Glycolysis: Lactate Levels Provide the Most Consistent Independent Risk Signal for the Low-Grade Serous/LMP Lineage

**Figure 4:**
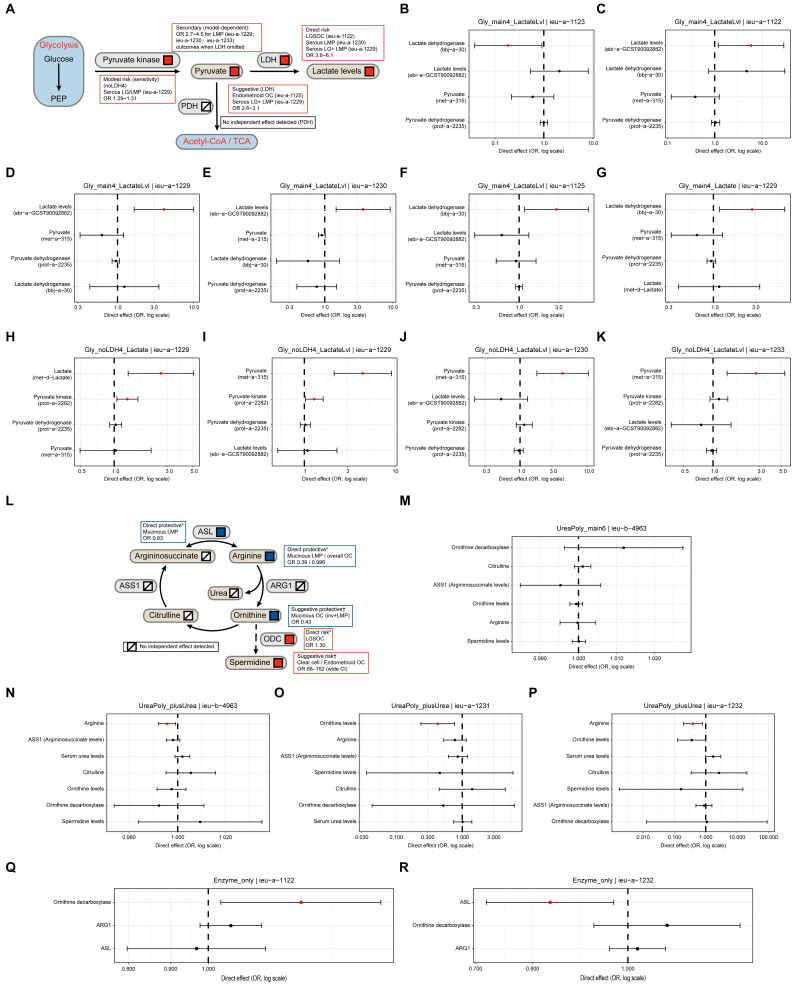
MVMR of terminal glycolysis and the urea cycle/polyamine axis across OC histotypes. (**A**) Terminal glycolysis schematic highlighting key metabolites included in MVMR and summarizing the direction/range of direct effects across model specifications. (**B**–**K**) Representative outcome forest plots for terminal glycolysis models, showing conditional direct effects of lactate (or lactate levels), pyruvate, LDH, PDH, and/or pyruvate kinase under different model specifications. (**L**) Urea cycle/polyamine schematic showing key metabolites and enzyme/protein proxy traits included in the models and summarizing highlighted direct effects. (**M**–**R**) Representative outcome forest plots for urea cycle/polyamine models, including the main model (UreaPoly_main6), the extended model adding serum urea (UreaPoly_plusUrea), and an enzyme/protein-only sensitivity model (Enzyme_only). Red points indicate nominal significance. Selected findings are highlighted in the text; full outcome-wide results, q values, and diagnostics are provided in Supplementary Figures S10–S17 and Supplementary Table S6. In the pathway schematics, black arrows indicate metabolic flow, and the dashed arrow indicates the polyamine branch from the urea-cycle/ornithine node. Red and blue squares/annotation boxes indicate risk-increasing and protective conditional direct-effect signals, respectively, whereas hatched squares indicate no independent conditional direct effect detected. Asterisks denote direct-effect signals from the prespecified primary model, and daggers denote suggestive or model-dependent signals supported mainly by sensitivity specifications. In the forest plots, dashed vertical lines indicate OR = 1, and arrows indicate truncated confidence intervals where applicable. Abbreviations: MVMR, multivariable MR; LDH, lactate dehydrogenase; PDH, pyruvate dehydrogenase; IVW, inverse-variance weighted; OR, odds ratio.

For terminal glycolysis, we specified a primary terminal-glycolysis MVMR model (Gly_main4_LactateLvl: lactate levels, pyruvate, LDH, and PDH) and conducted sensitivity analyses using an alternative lactate definition (Gly_main4_Lactate), a model including pyruvate kinase (Gly_main5), an LDH-excluded model (Gly_noLDH4), and a reduced branch/enzyme model (Gly_branch3_Pyr_LDH_PDH) (Supplementary Figures S10–S14; Supplementary Table S6). In the primary models (Figure 4A–K), LDH showed nominal conditional direct associations with IMOC (OR = 0.19, 95% CI 0.04–0.92; *p* = 0.038) and endometrioid OC (OR = 2.57, 95% CI 1.01–6.57; *p* = 0.048), suggesting potential histotype-dependent roles of lactate-related enzymes (Figure 4B,F). Within the same module, lactate levels provided the most consistent positive conditional direct-effect signal, with associations observed for LGSOC (OR = 6.05, 95% CI 1.41–25.97; *p* = 0.015), serous(LG + LMP) OC (OR = 4.04, 95% CI 1.67–9.76; *p* = 0.0019), and serous LMP-OC (OR = 3.61, 95% CI 1.27–10.27; *p* = 0.016) (Figure 4C–E; Supplementary Table S6). Alternative specifications yielded additional signals, but these were less stable overall and are therefore presented as exploratory (Figure 4H–K; Supplementary Figures S10–S14; Supplementary Table S6).

#### 2.3.4. Urea-Cycle/Polyamine Metabolism: Arginine, ASL, and ODC Show Suggestive Histotype-Specific Conditional Direct Effects

For urea-cycle/polyamine metabolism, we evaluated a prespecified urea-cycle/polyamine primary model (UreaPoly_main6), an expanded model including serum urea (UreaPoly_plusUrea), and an enzyme/protein-proxy-only model (Enzyme_only) as sensitivity specifications (Supplementary Figures S15–S17; Supplementary Table S6). In the UreaPoly_plusUrea model (Figure 4L–R), arginine showed a nominal mild protective effect in UK Biobank overall OC (OR = 0.996, 95% CI 0.992–0.999; *p* = 0.027) (Figure 4M) and a nominal protective conditional direct effect for mucinous LMP-OC (OR = 0.39, 95% CI 0.19–0.78; *p* = 0.0166) (Figure 4P). In the Enzyme_only model, ODC showed a nominal risk-increasing association with LGSOC (OR = 1.30, 95% CI 1.04–1.62; *p* = 0.0286) (Figure 4Q), whereas ASL showed a nominal protective association with mucinous LMP-OC (OR = 0.83, 95% CI 0.72–0.97; *p* = 0.021) (Figure 4R).

Taken together, pathway-restricted MVMR compressed the pathway-tracing results into three prioritized conditional-direct-effect themes—tryptophan and the low-grade/borderline serous lineage, lactate levels and the low-grade/borderline serous lineage, and urea/polyamine nodes and mucinous LMP/LGSOC—while highlighting that support was the strongest for the aromatic amino-acid module and more suggestive in the other modules (Figure 3 and Figure 4; Supplementary Table S6). These prioritized nodes provided a focused biological framework for subsequent translational analyses of glucose-lowering drug targets and multi-layer evidence integration.

### 2.4. Integrating Drug-Target MR, Colocalization, Triangular Evidence, and Mediation

**Figure 5:**
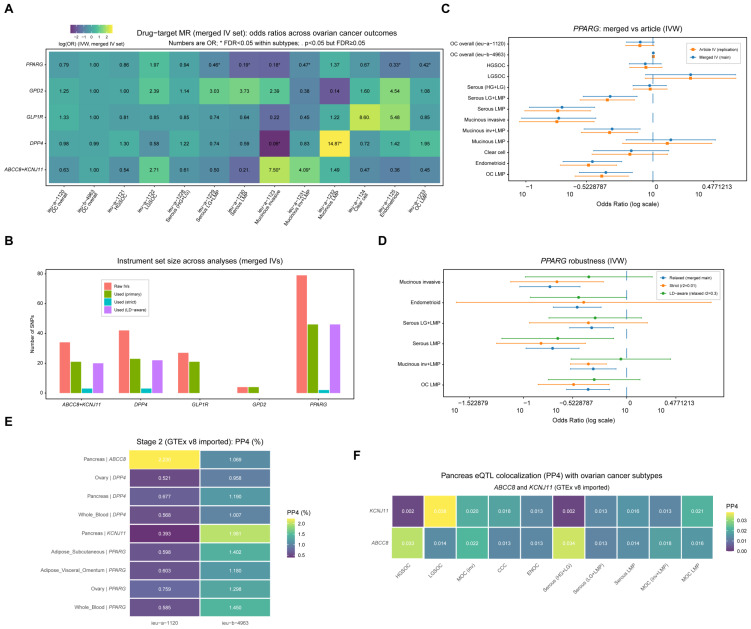
MR and genetic colocalization of glucose-lowering drug targets with OC outcomes. (**A**) Target MR landscape heatmap (target × outcome/histotype; merged cis-IV primary analysis). Color indicates log(OR), with OR values printed in cells; “*” denotes FDR-significant associations and “.” denotes nominal significance with FDR ≥ 0.05. (**B**) Instrument overview. The number of SNP instruments retained per target is shown across raw/primary/strict/LD-aware stages. (**C**) *PPARG* results comparing the merged primary analysis with the article IV replication. (**D**) *PPARG* robustness across relaxed merged, strict independent, and LD-aware correlated-SNP MR settings. (**E**) Colocalization PP4 heatmap. Posterior probability (PP4) of a shared causal variant between target eQTL signals and OC GWAS signals is shown across tissues. (**F**) Pancreas eQTL colocalization PP4 heatmap for *ABCC8/KCNJ11* across OC histotypes. Full MR results are provided in Supplementary Table S7; colocalization results in Supplementary Tables S8 and S9; sensitivity analyses (strict and LD-aware models) in Supplementary Table S10. Dashed vertical lines in the forest plots indicate OR = 1. Abbreviations: eQTL, expression quantitative trait locus; PP4, posterior probability for a shared causal variant in colocalization.

**Figure 6:**
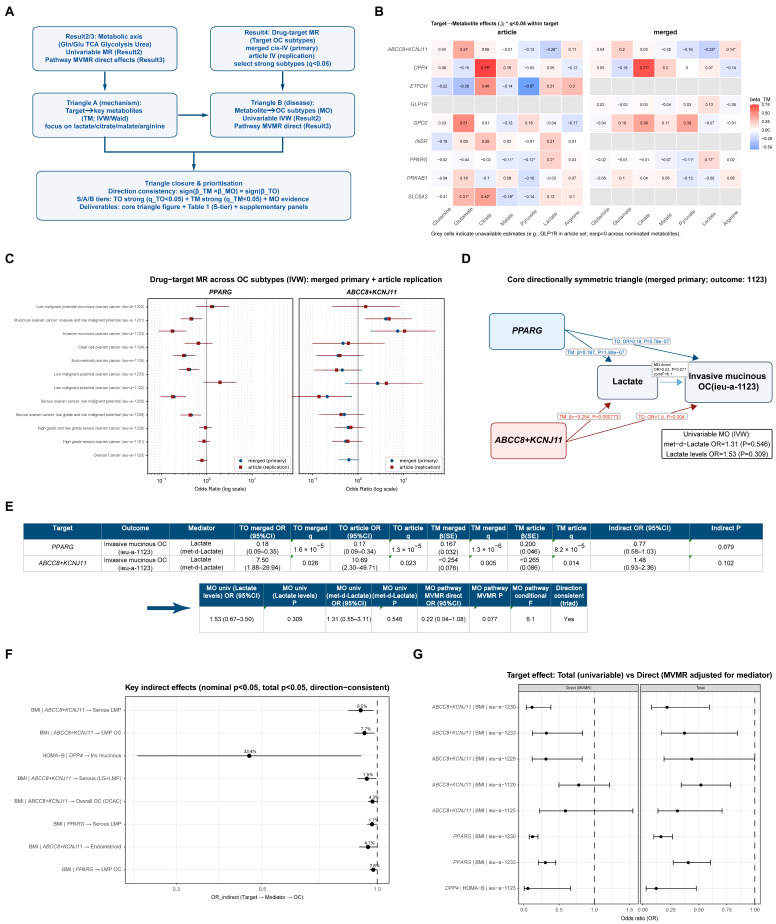
“Target–metabolite–outcome” triangular evidence and mediation analysis linking drug targets, the lactate/glycolysis axis, and OC histotypes. (**A**) Workflow for assembling triangular evidence across Target → Metabolite (TM), Metabolite → Outcome (MO), and Target → Outcome (TO), followed by prioritization based on directional consistency and statistical support. (**B**) TM heatmap. Target → metabolite effects (β) are shown for the merged primary analysis and the article IV replication; asterisks indicate within-target FDR significance. (**C**) TO forest plot. Effects of key targets are shown across OC outcomes/histotypes, comparing merged and article IV estimates. (**D**) Directionally symmetric “target–lactate–outcome” triangle for a representative outcome (ieu-a-1123), integrating TM, TO, MO (two lactate definitions in UVMR), and the conditional direct effect of lactate from the glycolysis MVMR. (**E**) Summary table of two high-grade “target–lactate–invasive mucinous OC (ieu-a-1123)” triangles, including TO/TM/MO evidence for *PPARG*–lactate–ieu-a-1123 and *ABCC8/KCNJ11*–lactate–ieu-a-1123, effect sizes (OR or β), significance (*p*/q), instrument counts, directional consistency, and indirect-effect estimates. (**F**) Key BMI-mediated indirect effects (OR, 95% CI), reporting mediation proportions where applicable. (**G**) Comparison of total effects (univariable) versus direct effects after BMI adjustment (MVMR) for the same key pairs. Key pairs are summarized in Table 2; complete two-step MR/MVMR and sensitivity analyses are provided in Supplementary Table S11. Grey cells indicate unavailable or non-estimable associations; arrows denote the direction of the target–metabolite–outcome relationship, and dashed lines indicate reference/null-effect thresholds where applicable. Abbreviations: TM, target-to-metabolite MR; MO, metabolite-to-outcome MR; TO, target-to-outcome MR.

Given the prominence of glycolysis-related nodes and glucose/insulin-regulatory themes in upstream analyses, we next tested whether genetic proxies for glucose-lowering drug targets influence OC risk and whether multi-layer evidence could connect target signals to the metabolic axis identified above (Figure 5 and Figure 6).

#### 2.4.1. Drug-Target MR Prioritizes *PPARG* and Identifies Additional Histotype-Dependent Signals for *DPP4*, *ABCC8/KCNJ11*, and *SLC5A2*

In the primary merged cis-IV analysis, drug-target MR highlighted *PPARG* as the most consistent protective target, while *DPP4*, *ABCC8/KCNJ11*, and *SLC5A2* showed more histotype-dependent and IV-definition-sensitive associations (Figure 5A,B; Supplementary Table S7). Within-target FDR-significant associations in the primary merged analysis are indicated in Figure 5A and detailed in Supplementary Table S7. Among the merged-analysis associations, *PPARG* was associated with reduced risk of serous LMP (OR = 0.19, 95% CI 0.11–0.35; *p* = 4.13 × 10^−8^), IMOC (OR = 0.18, 95% CI 0.09–0.35; *p* = 5.78 × 10^−7^), and endometrioid OC (OR = 0.33, 95% CI 0.19–0.56; *p* = 4.77 × 10^−5^) (Supplementary Table S7). By contrast, *DPP4* showed divergent histotype-specific associations, *ABCC8/KCNJ11* showed a risk-increasing association with IMOC under merged instruments, and *SLC5A2* showed a positive association with endometrioid OC under article instruments (Figure 5A; Supplementary Table S7). Overall, sensitivity analyses suggested that *PPARG* was comparatively robust, whereas several non-*PPARG* signals were more dependent on IV definition and LD handling (Figure 5C,D; Supplementary Figure S18A,C; Supplementary Table S10).

#### 2.4.2. Two-Stage Cis Colocalization Shows Limited Overall Support for Shared Causal Variants

We performed two-stage cis colocalization within ±500 kb windows (Stage 1: eQTLGen whole blood; Stage 2: GTEx v8 tissues) (Supplementary Figure S18D–F). Overall colocalization support was limited: top signals typically had PP4 around 0.02–0.04, far below commonly used thresholds for strong colocalization (e.g., PP4 > 0.8) (Figure 5E,F; Supplementary Tables S8 and S9). These results suggest that, in the current eQTL and OC datasets, evidence for a shared causal variant between target expression and OC risk is generally weak, despite the causal nominations from drug-target MR.

#### 2.4.3. Triangular Evidence Supports Directionally Coherent Target–Lactate–IMOC Patterns

To connect pathway MR with drug-target MR, we assembled triangular evidence across target-to-metabolite (TM), metabolite-to-outcome (MO), and target-to-outcome (TO) edges (Figure 6A). In IMOC (ieu-a-1123), *PPARG* and *ABCC8/KCNJ11* showed directionally coherent target–lactate–outcome patterns involving the same metabolic node (lactate) (Figure 6B–E). Using merged cis-IVs, *PPARG* was positively associated with lactate (beta = 0.167; *p* = 1.90 × 10^−7^) and inversely associated with IMOC risk (OR = 0.18; *p* = 5.78 × 10^−7^). Conversely, *ABCC8/KCNJ11* was inversely associated with lactate (beta = −0.254; *p* = 7.73 × 10^−4^) and positively associated with IMOC risk (OR = 7.50; *p* = 0.0043) (Figure 6B–E). The TM edges showed concordant directions under the article-IV sensitivity definition (Figure 6B), supporting robustness to the IV definition.

In MO analyses, lactate showed no clear association with IMOC in UVMR (met-d-Lactate: OR = 1.31; *p* = 0.546; lactate levels: OR = 1.53; *p* = 0.309) (Supplementary Figure S19B). After accounting for correlated glycolysis nodes in pathway-restricted MVMR, the conditional direct effect of lactate shifted toward a suggestive protective direction for IMOC (OR = 0.22; *p* = 0.077; conditional F = 6.1) (Figure 6D). Collectively, these analyses provide directional support for a lactate/glycolysis-centered IMOC pattern rather than a formally confirmed closed causal triangle.

#### 2.4.4. Mediation Analysis: BMI Shows a Consistent Risk-Increasing Direction, but Estimated Mediated Proportions of Target Effects Are Generally Small

Two-step MR supported a consistent risk-increasing direction for BMI in OC (Supplementary Figure S19C,D; Supplementary Table S11). However, evidence that drug-target effects were predominantly mediated through BMI was limited: estimated mediation proportions for key target–BMI–outcome chains were generally small (approximately 1.7–7.7%) (Figure 6F; Table 2), and many indirect effects were not statistically significant. Consistently, in BMI-adjusted MVMR validation, direct target effects were largely retained with limited attenuation (Figure 6G; Table 2).

An exception was observed for *DPP4*–IMOC, where the analysis showed nominal evidence of partial mediation via HOMA-B (indirect OR = 0.46; *p* = 0.025; mediation proportion approximately 33%), although this chain was sensitive to instrument availability and threshold settings (Figure 6F,G; Supplementary Figure S19E,F; Supplementary Table S11).

Overall, the integrated analyses prioritize *PPARG* as the most consistent protective target across IV definitions, indicate limited colocalization support, provide directional support for a *PPARG/ABCC8-KCNJ11*–lactate–IMOC pattern, and suggest that BMI explains only a limited fraction of the target–OC associations (Figure 5 and Figure 6; Table 2; Supplementary Figures S18 and S19; Supplementary Tables S7–S11).

### 2.5. Experimental Interrogation Supports the Directionally Symmetric Target–Lactate–IMOC Triangle in Mucinous Ovarian Cancer Models

#### 2.5.1. *PPARG* Defines the Protective Arm of the Target–Lactate–IMOC Triangle

**Figure 7:**
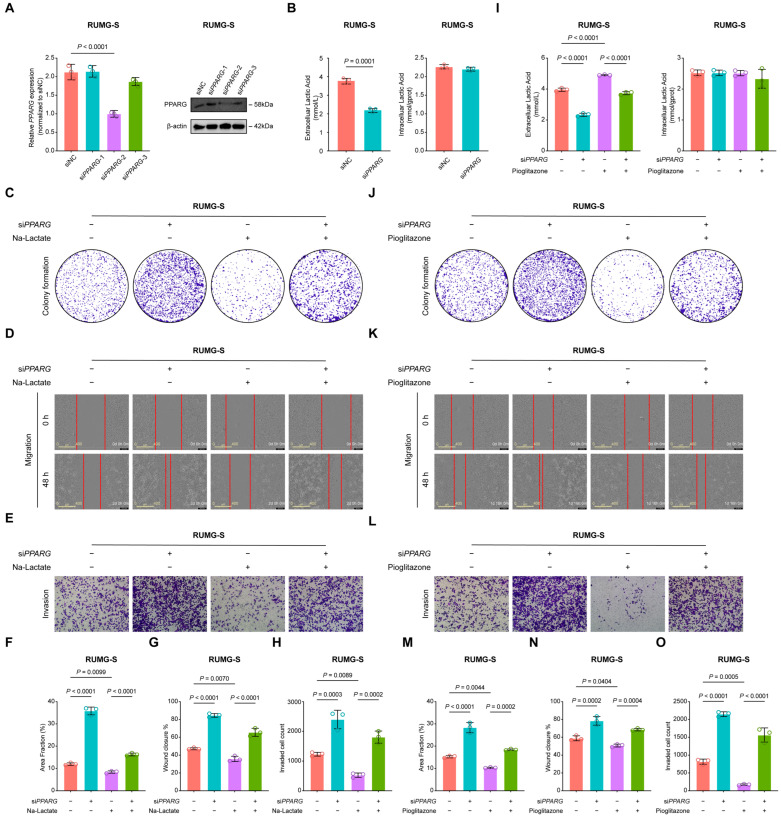
*PPARG* regulates lactate metabolism and malignant phenotypes in ovarian cancer cells. (**A**) RT–qPCR and immunoblot validation of siRNA-mediated *PPARG* knockdown efficiency in RMUG-S cells, with β-actin as the loading control. (**B**) Extracellular and intracellular lactate levels in RMUG-S cells following *PPARG* silencing. (**C**–**H**) Changes in malignant phenotypes in RMUG-S cells with *PPARG* silencing in the presence or absence of exogenous lactate supplementation, including colony formation ((**C**), with quantification shown in (**F**)), wound-healing migration ((**D**), with quantification shown in (**G**)), and Transwell invasion ((**E**), with quantification shown in (**H**)). (**I**) Extracellular and intracellular lactate levels in RMUG-S cells with *PPARG* silencing in the presence or absence of 10 μM pioglitazone. (**J**–**O**) Changes in malignant phenotypes in RMUG-S cells with *PPARG* silencing in the presence or absence of 10 μM pioglitazone, including colony formation ((**J**), with quantification shown in (**M**)), wound-healing migration ((**K**), with quantification shown in (**N**)), and Transwell invasion ((**L**), with quantification shown in (**O**)). Data are presented as mean ± SD from *n =* 3 independent experiments. Circles in the quantitative plots indicate individual independent experiments/replicates, and red vertical lines in the wound-healing images indicate the cell-front boundaries of the scratch area used for migration assessment. Two-group comparisons were analyzed using a two-tailed Student’s *t* test, and multiple-group comparisons were analyzed using one-way ANOVA.

To experimentally interrogate the MR-prioritized target–lactate–IMOC triangle, we first focused on *PPARG* in the mucinous ovarian cancer cell line RMUG-S [25]. siRNA-mediated *PPARG* silencing was efficiently validated at both the mRNA and protein levels (Figure 7A). Among the tested siRNAs, si*PPARG*-2 showed the most consistent knockdown efficiency and was therefore used in the subsequent functional experiments. Consistent with the predicted metabolic direction, *PPARG* knockdown reduced extracellular lactate, whereas intracellular lactate showed only a modest change (Figure 7B). We next examined whether lactate acted as a shared metabolic node linking *PPARG* perturbation to malignant phenotypes. In colony formation assays, exogenous sodium lactate partially attenuated the increase induced by *PPARG* silencing (Figure 7C,F). Likewise, sodium lactate partly suppressed the enhanced wound-healing migration and Transwell invasion caused by *PPARG* knockdown (Figure 7D,E,G,H). Together, these findings link *PPARG* loss to a lactate-low, more malignant cellular state and support a *PPARG*–lactate–malignant phenotype relationship.

To further probe the tractability of this arm, we next defined the pharmacological working condition for pioglitazone, a PPARγ agonist [26]. CCK-8 dose–response analysis in RMUG-S cells identified 10 μM for 72 h as the condition for follow-up assays (Figure S20A). Under this condition, pioglitazone restored extracellular lactate in *PPARG*-silenced cells, with little apparent change in intracellular lactate (Figure 7I). This was accompanied by attenuation of the enhanced colony formation (Figure 7J,M), wound closure (Figure 7K,N), and Transwell invasion (Figure 7L,O) induced by *PPARG* silencing. These data further support *PPARG* as a protective node that constrains malignant phenotypes through a lactate-linked metabolic axis.

We then extended this analysis to additional ovarian cancer models. In the mucinous ovarian cancer cell line MCAS [27], pioglitazone increased Incucyte-assessed cytotoxicity in a dose-dependent manner (Figure S20B,D), reduced Transwell invasion (Figure S20C,E), and increased extracellular lactate levels (Figure S20F). By contrast, the non-mucinous OVCAR8 line [28] showed only modest changes in cytotoxicity (Figure S20G,I), invasion (Figure S20H,J), and extracellular lactate (Figure S20K) under matched conditions. Together, these results position *PPARG* as the protective arm of the target–lactate–IMOC triangle, with a more coherent phenotypic effect in mucinous ovarian cancer models.

#### 2.5.2. *ABCC8/KCNJ11* Defines the Opposing Arm of the Target–Lactate–IMOC Triangle

**Figure 8:**
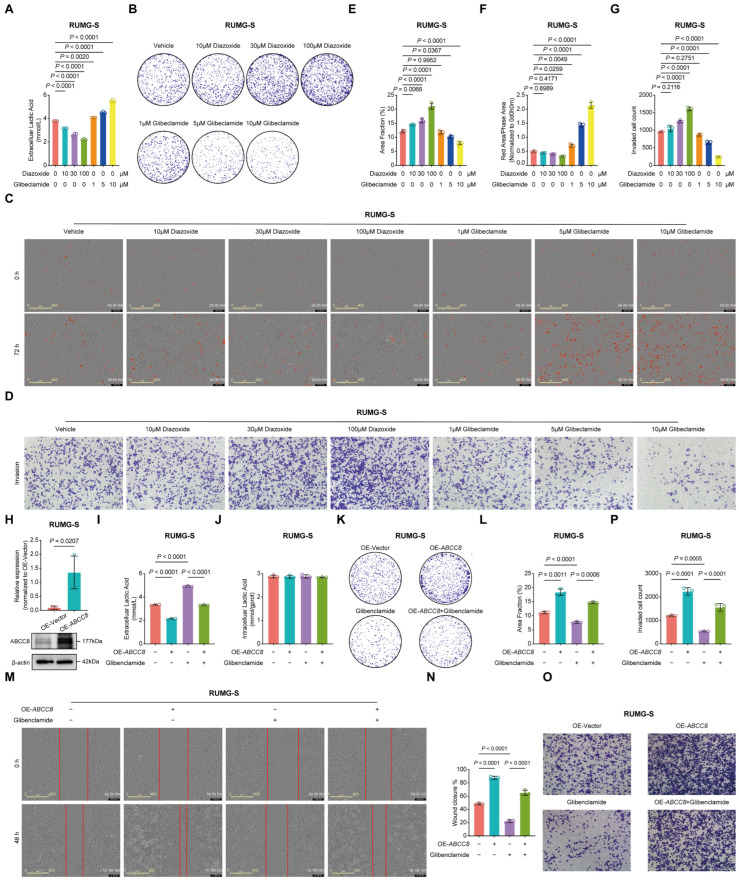
*ABCC8/KCNJ11* (the KATP complex) regulates lactate metabolism and malignant phenotypes in ovarian cancer cells. (**A**) Extracellular lactate levels in the mucinous ovarian cancer cell line RMUG-S following treatment with diazoxide (a KATP opener) or glibenclamide (a KATP inhibitor). (**B**–**G**) Changes in malignant phenotypes in RMUG-S cells following diazoxide or glibenclamide treatment, including colony formation ((**B**), with quantification shown in (**E**)), Incucyte-assessed cytotoxicity ((**C**), with quantification shown in (**F**)), and Transwell invasion ((**D**), with quantification shown in (**G**)). (**H**) RT–qPCR and immunoblot validation of *ABCC8* overexpression efficiency in RMUG-S cells. (**I**,**J**), Extracellular and intracellular lactate levels in RMUG-S cells with *ABCC8* overexpression in the presence or absence of glibenclamide. (**K**–**P**) Changes in malignant phenotypes in RMUG-S cells with *ABCC8* overexpression in the presence or absence of glibenclamide, including colony formation ((**K**), with quantification shown in (**L**)), wound-healing migration ((**M**), with quantification shown in (**N**)), and Transwell invasion ((**O**), with quantification shown in (**P**)). Data are presented as mean ± SD from *n =* 3 independent experiments. Circles in the quantitative plots indicate individual independent experiments/replicates, and red vertical lines in the wound-healing images indicate the cell-front boundaries of the scratch area used for migration assessment. Two-group comparisons were analyzed using a two-tailed Student’s *t* test, and multiple-group comparisons were analyzed using one-way ANOVA.

To interrogate the same target–lactate–IMOC triangle from the opposite regulatory direction, we next examined *ABCC8/KCNJ11*, the ATP-sensitive potassium (KATP) complex, in RMUG-S cells [29]. Pharmacological opening of KATP with diazoxide decreased extracellular lactate, whereas inhibition with glibenclamide increased it (Figure 8A) [30]. Concordant changes were observed at the phenotypic level: diazoxide enhanced colony formation (Figure 8B,E), reduced Incucyte-assessed cytotoxicity (Figure 8C,F), and increased Transwell invasion (Figure 8D,G), whereas glibenclamide showed the opposite pattern. These reciprocal effects support an inverse relationship between KATP activity and the lactate-linked malignant phenotype.

To complement the pharmacological perturbation, we further overexpressed *ABCC8* in RMUG-S cells. *ABCC8* overexpression was validated at both the mRNA and protein levels by RT-qPCR and Western blotting (Figure 8H). In *ABCC8*-overexpressing cells, extracellular lactate was reduced and was restored by glibenclamide (Figure 8I), whereas intracellular lactate showed no marked shift across groups (Figure 8J). Functionally, *ABCC8* overexpression increased colony formation (Figure 8K,L), accelerated wound-healing migration (Figure 8M,N), and enhanced Transwell invasion (Figure 8O,P), and each of these changes was attenuated by glibenclamide. Collectively, these bidirectional genetic and pharmacological data place *ABCC8/KCNJ11* opposite to *PPARG* within the target–lactate–IMOC triangle, supporting a model in which *PPARG* and KATP signaling drive opposing lactate-linked metabolic states that converge on malignant behavior in mucinous ovarian cancer cells.

Taken together, these findings extend the MR-prioritized target–lactate–IMOC triangle into an experimentally supported framework in mucinous ovarian cancer. *PPARG* and *ABCC8/KCNJ11* converged on lactate as a shared metabolic node but regulated it in opposite directions, corresponding to a protective arm and an opposing pro-malignant arm, respectively. These data therefore connect the genetic and experimental results into a coherent histotype-specific model.

## 3. Discussion

This study applied a histotype-stratified metabolome-wide Mendelian randomization (MR) framework to ovarian cancer outcomes and translated dispersed metabolomic signals into a more interpretable and translationally relevant causal chain. Moving beyond single-metabolite nomination, we integrated pathway tracing, pathway-restricted multivariable MR, drug-target MR, mediation analysis, and experimental interrogation to connect metabolic signals with potentially actionable metabolic targets. We observed pronounced histotype dependence of the metabolic causal signals in ovarian cancer: signals that appear scattered at the single-metabolite level could be organized along a continuous “amino-acid/energy–nitrogen metabolism axis”, within which the glycolysis-end module, particularly lactate/LDH-related traits, most effectively linked metabolic nodes to drug-target genetic evidence. At the target level, genetically proxied *PPARG* showed a relatively consistent protective direction across multiple outcomes, whereas *ABCC8/KCNJ11* showed the opposite direction in IMOC and, together with lactate-related traits, formed a directionally symmetric target–lactate–IMOC triangle. These findings are consistent with the view that epithelial ovarian cancer is not a single entity but a group of histotypes with distinct origins, molecular characteristics, and risk-factor profiles.

A large body of observational metabolomics literature, including prospective and prediagnostic plasma metabolomics studies, has suggested that ovarian cancer risk is linked to perturbations in amino-acid and energy metabolism, yet findings have not been fully consistent across cohorts. This inconsistency is partly expected: when the case mix, particularly the histotype distribution, differs across studies, the observed metabolomic signature is also more likely to diverge. For example, a prospective analysis in the Nurses’ Health Studies identified circulating plasma metabolite patterns associated with epithelial ovarian cancer risk and suggested histotype-dependent associations for some metabolites [1,6,31]. Our genetic results support the interpretation that the heterogeneity seen in prior studies is unlikely to be entirely attributable to confounding or reverse causation; instead, part of it may reflect genuine etiologic differences across histotypes. Organizing these signals into a mechanistic axis that integrates carbon/energy metabolism with nitrogen handling helps convert dispersed “point nominations” into biologically interpretable modules. This interpretation also aligns with canonical views of cancer metabolic reprogramming, in which imbalances in glucose and amino-acid acquisition and increased nitrogen demand are expressed as coordinated shifts across interconnected pathways rather than as the isolated effect of a single circulating metabolite [8,9].

At the drug-target level, *PPARG* was the glucose-lowering target with the most consistent protective direction in this study, whereas *ABCC8/KCNJ11* showed the opposite direction in IMOC. This direction is broadly consistent with prior experimental evidence showing that PPARγ activation can suppress ovarian cancer cell proliferation and promote apoptosis, although the effects are model-dependent and may not be fully mediated by PPARγ itself. Our experimental data further support the proposed “target–lactate–IMOC” loop and indicate that lactate is not merely an outcome-associated metabolite, but rather a key functional node linking target perturbation to phenotypic change in tumor cells [10,32]. This also helps explain why the lactate → IMOC association was not evident in univariable MR, yet moved closer to the direction required for “triangle closure” under glycolysis-pathway MVMR with correlation adjustment. A more coherent interpretation is that lactate does not represent a single causal exposure sufficient to define the whole chain on its own, but rather a key measurable component of the broader glycolysis-end state. Its biological meaning becomes clearer only when it is interpreted within the network context formed by other correlated metabolic nodes. Taken together, the genetic and experimental results suggest that what is biologically most relevant is not lactate in isolation, but whether a lactate-centered glycolysis-end state is systematically remodeled under different target perturbations and, in turn, affects malignant behavior in mucinous ovarian cancer.

This framework also helps explain several time-dependent and model-dependent features of the experimental data. The clearer inhibitory effect of pioglitazone in RMUG-S cells only becomes evident at 72 h is biologically plausible, because PPARγ is a ligand-activated nuclear receptor/transcription factor, and its anti-proliferative or pro-apoptotic effects usually depend on downstream transcriptional reprogramming; accordingly, short-term readouts are more susceptible to transient metabolic adaptation [33,34]. In addition, the clearer dose-dependent response in MCAS, together with the relatively limited effect in non-mucinous OVCAR8, further suggests that this loop may be more closely aligned with the metabolic context of the mucinous lineage rather than representing a uniform mechanism across all ovarian cancer models. Accordingly, future validation should focus on integrated readouts of the glycolysis-end state and determine, in mucinous-lineage models, whether perturbation of *PPARG* or KATP signaling is accompanied by coordinated changes in this terminal metabolic state and its downstream phenotypes. Such a validation strategy would more effectively translate genetic prioritization into measurable, targetable, and experimentally testable mechanistic hypotheses.

The BMI mediation analyses further constrained how the target signals should be interpreted. Prior large-scale MR studies support a causal role of adult BMI in ovarian cancer risk and suggest heterogeneity across histotypes, with potentially stronger effects in non-high-grade serous subtypes [35]. In our framework, however, the proportion of *PPARG*/KATP target effects mediated through BMI was generally small, and the direct effects of the targets largely persisted in models that accounted for BMI. Together with MR evidence linking insulin-related traits, such as impaired insulin secretion, to ovarian cancer risk, we suggest that genetically proxied target perturbation is unlikely to be merely a surrogate for adiposity; instead, it may implicate metabolic programs not fully explained by adiposity alone, including β-cell/insulin secretion pathways and immunometabolic states [36]. Accordingly, treating BMI as the sole mediator in downstream clinical and mechanistic validation may underestimate or misattribute target-relevant biology; a more appropriate approach is to maintain histotype stratification while incorporating insulin-related measures and lactate/LDH end-state readouts that more closely represent the proposed metabolic axis.

From a translational perspective, this study provides a conservative genetic prioritization map rather than an immediately actionable prescribing recommendation. Its value lies not only in genetically prioritizing *PPARG*-related perturbation as a potentially protective direction, but also in identifying which “histotype–mechanism chains” merit priority validation in cohorts and experimental systems [20]. The current experimental findings make this prioritization more concrete: mucinous settings appear to be a rational first context for testing the target–lactate–IMOC chain, and joint readouts of lactate/LDH-end states together with their neighboring network components are likely to reflect the relevant metabolic state more effectively than a single short-term lactate measurement. Important limitations, however, remain. The screening stage is exploratory, and some metabolite associations remain nominal; pathway-restricted MVMR may be affected by conditional weak instruments and collinearity; sample overlap and ancestry differences between exposure and outcome datasets may introduce bias; and colocalization analyses remain constrained by tissue relevance and statistical power. At the experimental level, the current validation evidence is still derived primarily from in vitro models. Most importantly, MR reflects lifelong genetic perturbation and cannot be directly extrapolated to drug dose, treatment timing, or off-target effects [14,15,37]. For these reasons, the present findings are better viewed as a starting point for staged validation and trial design rather than as a direct basis for clinical intervention. A more appropriate next step is therefore tiered: histotype-stratified pharmacoepidemiologic evaluation in exposed populations, biomarker studies focused on lactate/LDH-end states and their surrounding network context, and experimental perturbation of *PPARG*/KATP with parallel monitoring of glycolysis-end and immunometabolic consequences.

## 4. Methods

### 4.1. Study Design and Reporting Framework

We conducted a multi-stage, two-sample Mendelian randomization (MR) study to evaluate histotype-specific metabolic determinants of ovarian cancer (OC) and to connect these signals to potentially actionable glucose-lowering drug targets. Except for the cell-based experiments described in Section 4.14, Section 4.15 and Section 4.16, all analyses used externally generated summary-level GWAS/eQTL association statistics rather than individual-level data. The workflow was organized sequentially as follows. First, we performed metabolome-wide nomination screening of 1400 circulating plasma metabolic traits against two independent overall OC outcomes, followed by post-MR KEGG pathway enrichment to identify convergent biochemical themes (Section 4.5). Second, we translated these nominated pathway themes into histotype-stratified pathway-guided univariable MR follow-up analyses along a predefined amino acid/energy–nitrogen axis and applied Bayesian weighted MR (BWMR) as a robustness estimator (Section 4.6 and Section 4.7). Third, we used pathway-restricted multivariable MR (MVMR) to estimate conditional direct effects within correlated metabolic modules (Section 4.8). Fourth, we evaluated cis-proxied glucose-lowering drug targets using drug-target MR, two-stage cis colocalization, target–metabolite–outcome evidence integration, and two-step summary-data mediation with paired MVMR robustness analysis (Section 4.9, Section 4.10, Section 4.11 and Section 4.12). Finally, we performed newly generated laboratory experiments in ovarian cancer cell models to interrogate the prioritized *PPARG/ABCC8/KCNJ11*–lactate–IMOC hypothesis (Section 4.14, Section 4.15 and Section 4.16). Reporting followed the STROBE-MR guideline [18], and detailed information on data provenance, instrument construction, sensitivity analyses, multiplicity handling, and software is provided in the corresponding subsections: Table 1 and Supplementary Table S1.

This study was intentionally designed as a multi-stage two-sample MR investigation using externally generated GWAS/eQTL summary association datasets rather than de novo GWAS or individual-level regression analyses, consistent with standard MR methodology and reporting guidance; summary-data-specific limitations such as restricted assumption checking, possible sample overlap, and ancestry mismatch were considered in interpretation [15,18,21,38,39,40].

### 4.2. Data Sources, Data Level, and Phenotype Definitions

For the MR, mediation, and colocalization components of this study, all genetic association analyses were based on publicly available external summary-level data. We did not perform de novo GWAS, metabolome-wide association analyses, or any participant-level regression analyses. Except for the cell-based experiments described in Section 4.14, Section 4.15 and Section 4.16, all analyses in Section 4.2, Section 4.3, Section 4.4, Section 4.5, Section 4.6, Section 4.7, Section 4.8, Section 4.9, Section 4.10, Section 4.11 and Section 4.12 used externally generated summary-level association statistics. GWAS summary datasets were accessed through the IEU OpenGWAS platform (MR-Base infrastructure) [21], whereas the cis-eQTL summary datasets used for colocalization were obtained from eQTLGen and GTEx [41,42]. Accordingly, the original SNP–trait association estimates, phenotype definitions, genotype quality-control/imputation procedures, recruitment schemes, covariate-adjustment strategies, and source-study association models were generated by the original GWAS/eQTL investigators rather than by the authors. In the present study, we extracted, harmonized, and re-analyzed these published summary data within a multi-stage two-sample MR framework [18,21,39,40]. To make data provenance explicit, Table 1 and Supplementary Table S1 summarize for each dataset its data level, source/consortium, OpenGWAS ID (or source label), sample size, ancestry, phenotype definition/scale, and analytical role in the present study.

The metabolome-wide screening stage used 1400 circulating plasma metabolic traits from the genomic atlas of the plasma metabolome by Chen et al. [19], accessed through IEU OpenGWAS [21]. This panel comprised both metabolite concentrations and metabolite ratios; the full list of trait names, OpenGWAS/GCST IDs, sample sizes, ancestry, and measurement information is provided in Supplementary Table S2. In this manuscript, “metabolome-wide screening” refers to metabolome-wide Mendelian randomization screening across these pre-existing metabolite GWAS traits, rather than GWAS or metabolomic association analyses performed by the authors.

For ovarian cancer outcomes, we used two independent overall ovarian cancer GWAS summary datasets, from UK Biobank (ieu-b-4963) and the Ovarian Cancer Association Consortium (OCAC; ieu-a-1120), together with OCAC histotype- and malignant-potential-specific outcomes (e.g., HGSOC, LGSOC, IMOC, clear cell, endometrioid, serous LMP, mucinous LMP, and related combined phenotypes) (Table 1; Supplementary Table S1). We did not download or analyze individual-level UK Biobank, OCAC, or other cohort data, and we did not re-run logistic regression or any other participant-level outcome model. Therefore, all outcome effect estimates used in the MR analyses were inherited summary-level SNP–ovarian cancer association statistics from the source GWAS.

Additional external summary datasets were used for downstream analyses. Pathway-tracing exposures and pathway-restricted MVMR included selected metabolites, lactate-related biomarkers, and enzyme/protein proxy traits chosen a priori according to their positions along the predefined amino acid/energy–nitrogen axis and the availability of suitable summary data (Table 1; Supplementary Tables S1, S5 and S6). Drug-target MR used cis-acting genetic proxies for glucose-lowering drug targets, derived from published cis-instrument sources and from a blood-glucose GWAS used for online cis-IV extraction. Two-step mediation analyses used publicly available summary statistics for BMI, WHR, fasting insulin, and HOMA-B, and two-stage colocalization used cis-eQTL summary statistics from eQTLGen and GTEx. Because this study reused external summary statistics from multiple sources, dataset-specific limitations such as possible sample overlap, ancestry mismatch, and heterogeneity in source phenotype definitions and modeling strategies are acknowledged explicitly and discussed in Section 4.13.

### 4.3. Instrument Selection, LD Clumping, and Harmonization from External Summary Statistics

Using the external summary-level datasets described in Section 4, we extracted SNP–exposure association estimates (β_GX and SE_GX_) and SNP–outcome association estimates (β_GY and SE_GY_) from the external summary datasets described in Section 4.2, Table 1, and Supplementary Table S1, and used these harmonized summary statistics as the inputs for two-sample MR analyses [18,21,38,39]. Thus, Section 4.3 and Section 4.4 did not involve fitting new participant-level linear regression, logistic regression, or survival models. The source-study response variables (Y), genetic predictors (X), and upstream covariate-adjustment models were defined by the original GWAS investigators and are now summarized at the dataset/family level in Supplementary Table S1.

SNP instruments for metabolite and biomarker exposures entering UVMR and BWMR were selected from OpenGWAS using a unified pipeline implemented in R (version 4.5.2) with the ieugwasr (version 1.1.0) and TwoSampleMR (version 0.6.29) packages [21]. We first selected variants associated with the exposure at *p* < 5 × 10^−8^ and then performed LD clumping against the European reference panel from the 1000 Genomes Project [43] using r^2^ < 0.01 within 1000 kb. When fewer than three SNPs remained after clumping at *p* < 5 × 10^−8^, the threshold was relaxed to *p* < 5 × 10^−6^ or *p* < 5 × 10^−5^ to retain an estimable instrument set; the threshold actually used for each analysis is reported in the corresponding supplementary tables, including Supplementary Tables S1, S5–S7, S10 and S11. Instrument strength was screened using the approximate F statistic, F = (β_GX/SE_GX_)^2^, and analyses with potential weak-instrument concerns were interpreted cautiously [44].

Exposure and outcome datasets were then harmonized to align effect alleles and directions. Palindromic variants were handled conservatively, and incompatible alleles were excluded. For analyses that required strict SNP alignment across multiple downstream datasets, particularly the two-step mediation analyses, LD proxies were searched when an instrument SNP was unavailable in a downstream dataset (r^2^ ≥ 0.8), and all proxy substitutions were documented in Supplementary Table S11. For pathway-restricted MVMR and target-specific cis-instrument construction, instrument-building rules differed from the general UVMR/BWMR pipeline and are therefore described separately in Section 4.8, Section 4.9 and Section 4.12.

### 4.4. Summary-Data Univariable Two-Sample MR Estimation and Sensitivity Analyses

After harmonization, causal effects were estimated directly from the summary-level SNP–exposure and SNP–outcome association coefficients rather than from participant-level observations. For a given SNP *i*, the SNP-specific causal estimate was obtained by the Wald ratio (β_GY,i_/β_GX,i_); when multiple independent SNPs were available, SNP-specific estimates were combined using inverse-variance weighting (IVW), which served as the primary univariable two-sample MR estimator [38]. Single-SNP instruments were analyzed using the Wald ratio. Here, the present-study exposure was the selected metabolite or biomarker trait, and the present-study outcome was ovarian cancer risk (overall or histotype-specific) represented by the source-study summary association estimates. For binary ovarian cancer outcomes, effect estimates were reported as odds ratios (ORs) per genetically predicted increase in the exposure on its original GWAS scale.

Sensitivity analyses were performed where the number of instruments allowed, using TwoSampleMR (version 0.6.29) and MRPRESSO (version 1.0) in R. These included MR-Egger regression [45], weighted median [46], and mode-based estimators [47]; Cochran’s Q for between-SNP heterogeneity; the MR-Egger intercept test for directional horizontal pleiotropy; MR-PRESSO for outlier detection/correction [48]; and leave-one-out analyses. To avoid misleading or underpowered diagnostics, these procedures were applied only when the minimum instrument-number requirements were met (typically at least three SNPs, with MR-PRESSO generally requiring more instruments). Because LD clumping alone cannot guarantee the independence and exclusion-restriction assumptions, associations were interpreted by jointly considering instrument strength, concordance across estimators, heterogeneity/pleiotropy diagnostics, and downstream triangulation analyses [18,38,45,46,47,48].

### 4.5. Metabolome-Wide Two-Sample MR Nomination Screening and Post-MR Pathway Enrichment

Section 4.5, Section 4.6, Section 4.7 and Section 4.8 were intentionally organized as a layered workflow rather than a set of interchangeable analyses. All analyses in these sections used the summary-data MR framework established in Section 4.3 and Section 4.4 and were performed in R (version 4.5.2; package versions listed in Section 4.13) [21,38,39], except the KEGG pathway analysis, which was conducted in MetaboAnalyst 5.0. Section 4.5 served as an unbiased nomination stage at the metabolome-wide level; Section 4.6 translated nominated pathways into histotype-stratified node-level follow-up analyses; Section 4.7 applied BWMR only as a robustness estimator for selected UVMR-positive pairs; and Section 4.8 estimated conditional direct effects within correlated metabolic modules. These steps therefore addressed different analytical questions and were interpreted sequentially.

As an exploratory discovery stage, we conducted a metabolome-wide two-sample MR screening analysis for each of the 1400 circulating plasma metabolic traits against two independent overall OC outcomes (UK Biobank: ieu-b-4963; OCAC: ieu-a-1120; Figure 1A). Instrument selection, LD clumping, harmonization, and primary causal estimation followed the procedures described in Section 4.3 and Section 4.4 [21,38]. To maintain a uniform screening framework across the full metabolite panel, candidate metabolites were nominated using the primary UVMR estimator at nominal significance (*p* < 0.05), with this stage intended for hypothesis generation rather than definitive causal confirmation. Screening results are reported in Supplementary Table S3.

To summarize whether metabolite-level nominated signals converged on biologically interpretable themes, we then performed pathway enrichment and topology analyses on the IVW-positive metabolite sets from each overall OC outcome separately. This pathway analysis was conducted in MetaboAnalyst using KEGG as the reference pathway database, and raw *p* values, Benjamini–Hochberg FDR-adjusted significance, pathway impact, and hit counts were reported [22,23,24]. Thus, pathway analysis served to organize dispersed metabolite-level signals into pathway-level biochemical themes for subsequent follow-up, rather than to generate causal effect estimates itself. Enrichment results are reported in Supplementary Table S4.

### 4.6. Pathway-Guided Histotype-Stratified Follow-Up UVMR (“Pathway-Tracing”)

The term “pathway-tracing” is used here as a study-specific label rather than as a distinct published MR estimator. It denotes the sequential follow-up of biologically connected pathway nodes after the metabolome-wide nomination stage, using the same summary-data UVMR framework as Section 4.4 but applied to histotype- and malignant-potential-stratified OC outcomes. Candidate nodes were selected a priori according to three criteria: (i) metabolites or pathways nominated in Section 4.5; (ii) upstream/downstream biological adjacency along the predefined amino acid/energy–nitrogen axis; and (iii) availability of suitable metabolite, biomarker, or enzyme/protein-proxy summary statistics in OpenGWAS (Table 1; Supplementary Table S1). In this study, the predefined amino acid/energy–nitrogen axis comprised amino acid-related nodes, TCA-cycle intermediates, glycolysis/lactate end-products and key enzymes, and urea-cycle/polyamine nodes, reflecting a pathway architecture relevant to tumor metabolic reprogramming.

Accordingly, Section 4.6 did not repeat the metabolome-wide screen. Instead, it converted the nominated pathway themes into mechanistically connected, histotype-stratified UVMR follow-up analyses. These analyses were implemented in R using ieugwasr and TwoSampleMR, with IVW/Wald ratio as the primary estimators and the sensitivity procedures described in Section 4.4 applied when the number of instruments permitted [21,38,39]. Complete pathway-tracing outputs are provided in Supplementary Table S5.

### 4.7. Bayesian Weighted MR (BWMR) as a Robustness Analysis

BWMR was not used as a second discovery screen. Rather, it was applied only to selected exposure–outcome pairs highlighted by the pathway-guided UVMR stage, in order to assess whether nominal UVMR signals remained directionally supported under a Bayesian estimator that can down-weight potentially invalid or weak instruments [49]. BWMR used the same harmonized summary association inputs and the same instrument sets as the corresponding UVMR analyses, and was implemented in R using the BWMR algorithm described by Zhao et al. [49].

The BWMR model was fitted using a variational EM algorithm with fixed hyperparameters: prior variance σ_0_^2^ = (10^6^)^2^, α = 100, maximum iterations = 5000, and convergence defined as relative evidence lower bound (ELBO) change <1 × 10^−6^. An association was labeled as “BWMR supported” only when the BWMR and IVW directions were concordant and BWMR *p* < 0.05. BWMR therefore served as a robustness layer on top of UVMR, rather than as an independent pathway-discovery stage.

### 4.8. Pathway-Restricted Multivariable MR (MVMR) for Conditional Direct Effects Within Correlated Modules

Whereas the UVMR analyses in Section 4.6 estimated the total effect of one pathway node at a time, MVMR was used here to answer a different question: whether a nominated node retained an association with the outcome after mutual adjustment for correlated neighboring exposures within the same biochemical module [50]. This distinction is particularly important in metabolomics, where metabolites and enzyme/protein proxies within a pathway often share genetic architecture and can generate overlapping UVMR signals. Section 4.8 was therefore designed as a downstream refinement step, not as a repetition of Section 4.6.

To balance biological coverage against instrument strength and multicollinearity, we prespecified low-dimensional, pathway-restricted models within each module and designated one primary model per module for main-text reporting, with alternative model specifications treated as robustness analyses rather than separate discovery experiments. Modules included aromatic amino acids, TCA-cycle intermediates, terminal glycolysis/lactate, and urea-cycle/polyamine metabolism (definitions and exposure composition in Supplementary Table S6). For each model, instruments were combined across exposures (union set), stringently LD clumped using the European 1000 Genomes reference panel (r^2^ < 0.001 within 10 Mb), and restricted to common SNPs with complete SNP–exposure associations for all included exposures. No proxy substitution was used for missing bx values in MVMR.

After allele harmonization across exposures and outcomes, conditional direct effects were estimated under the IVW-MVMR framework in R following the summary-data approach of Sanderson et al. [50]. Conditional F statistics were used to assess instrument strength, and weak-instrument models were interpreted cautiously. Because alternative exposure definitions within the same module can change both instrument composition and collinearity, these additional specifications were interpreted as sensitivity analyses to assess the stability of prioritized nodes. Full model outputs and diagnostics are reported in Supplementary Table S6.

### 4.9. Drug-Target MR Using Cis Genetic Proxies for Glucose-Lowering Targets

Section 4.9, Section 4.10, Section 4.11 and Section 4.12 constitute a target-centered translational layer of the study. Section 4.9 estimated the effects of genetically proxied glucose-lowering drug targets on ovarian cancer (OC) and selected metabolic nodes; Section 4.10 assessed whether target regulatory and OC association signals were consistent with a shared causal variant; Section 4.11 integrated target, metabolite, and outcome edges into study-specific evidence triangles; and Section 4.12 examined whether selected clinical mediators explained part of the target–outcome associations. All analyses in Section 4.9, Section 4.10, Section 4.11 and Section 4.12 used the external summary-level GWAS/eQTL datasets described in Section 4.2 [18,21,38] and formed the target-centered translational layer of the study.

#### 4.9.1. Targets and Cis-Instrument Definitions

To extend pathway-level metabolic findings toward actionable mechanisms, we next evaluated whether genetically proxied perturbation of glucose-lowering drug targets was associated with OC risk. We assessed a broader panel of glucose-lowering targets represented in the target MR landscape, including *PPARG*, *DPP4*, *GLP1R*, *ABCC8/KCNJ11*, *SLC5A2*, *INSR*, *GPD2*, and *ETFDH*, and subsequently prioritized targets showing stronger or more interpretable signals for downstream multi-layer integration (Table 1; Figure 5) [51].

Drug-target MR was performed within a cis-proxy framework, using variants in or near the target gene as genetic proxies for target perturbation relevant to glucose lowering [20,51]. To reduce dependence on a single instrumental-variable (IV) definition, we constructed three cis-instrument sets per target: (i) “article” cis-IVs curated from published drug-target MR studies [51]; (ii) “online” cis-IVs extracted from an external blood-glucose GWAS (OpenGWAS: ebi-a-GCST90025986); and (iii) “merged” cis-IVs created as the union of article and online sets, followed by target-specific LD handling. The present study inputs were summary-level SNP–blood glucose coefficients from the instrument-source GWAS, together with summary-level SNP–OC or SNP–metabolite coefficients from the corresponding outcome datasets; no participant-level blood-glucose or cancer data were analyzed in this section.

#### 4.9.2. Online Cis-IV Extraction

Genome-wide significant blood-glucose variants (*p* < 5 × 10^−8^) were extracted without initial clumping [52], and then LD clumped with relaxed parameters (clump_kb = 100; clump_r2 = 0.3; clump_p1 = clump_p2 = 5 × 10^−8^). Variants were subsequently restricted to the cis region of each target gene, defined as gene boundaries ±3 Mb (GRCh37/hg19 coordinates as specified in the extraction script) [27]. Online extraction was implemented in R using ieugwasr/OpenGWAS queries and custom scripts [21].

#### 4.9.3. Primary and Sensitivity Analyses

Primary drug-target MR analyses used the merged cis-IV set under relaxed LD handling to retain sufficient instrument density, with IVW as the main estimator and the Wald ratio for single-SNP sets. Within each target, multiplicity across OC outcomes/histotypes was controlled using the Benjamini–Hochberg FDR procedure [24]. Robustness analyses included: (i) strict independent cis-IV sets obtained by more stringent re-clumping of the merged set; and (ii) LD-aware correlated-SNP MR for relaxed cis-IV sets retaining correlated variants, using LD-aware IVW, LD-aware Egger, and LD-aware maximum-likelihood estimators implemented in the MendelianRandomization package with LD matrices referenced to the European 1000 Genomes panel [43,53]. Exact SNP composition and LD settings for the relaxed, strict, and LD-aware analyses are reported in Supplementary Tables S7 and S10. In addition to target → outcome analyses, the same cis-proxy framework was used for target → metabolite analyses, entering the triangular-evidence step (Section 4.11).

### 4.10. Two-Stage Cis Colocalization of Target Regulatory and Ovarian Cancer Signals

For target–outcome pairs emerging from the drug-target MR stage, we next evaluated whether target regulatory signals and OC GWAS signals were compatible with a shared causal variant using two-stage Bayesian cis colocalization. To assess whether a target regulatory signal and an OC GWAS signal were likely driven by the same causal variant, we performed two-stage Bayesian cis colocalization using coloc.abf within ±500 kb around each target gene [54]. Stage 1 used eQTLGen whole-blood cis-eQTLs as a prescreen [41], and Stage 2 used GTEx v8 cis-eQTLs across tissues for refinement [42]. Analyses were implemented in R using the coloc package (version 5.2.3; software version listed in Section 4.13). We matched SNPs by rsID, collapsed duplicated rsIDs by retaining the smallest *p* value, removed missing/non-finite values, and restricted minor allele frequencies to (0, 0.5). Posterior probabilities for hypotheses H0–H4 were estimated; PP4 (shared causal variant) was the primary metric, and PP4_over = PP4/(PP3 + PP4) was reported as an auxiliary diagnostic. Priors were set to p1 = 1 × 10^−4^, p2 = 1 × 10^−4^, and p12 = 1 × 10^−5^. This step was intended as an orthogonal shared-variant check for the drug-target MR results rather than as a second causal-effect estimator.

### 4.11. Study-Specific Target–Metabolite–Outcome Evidence Integration (“Triangular Evidence”)

Section 4.11 was a study-specific evidence-integration step rather than a standalone published estimator [55]. To connect the upstream metabolic axis with drug-target findings, we then assembled target–metabolite–outcome evidence triangles by integrating three directed edges within a common summary-data framework: target-to-outcome (TO) edges from Section 4.9, metabolite-to-outcome (MO) edges from the pathway-tracing UVMR and pathway-restricted MVMR stages (Section 4.6, Section 4.7 and Section 4.8), and target-to-metabolite (TM) edges estimated using the same cis-acting target instruments against publicly available metabolite or biomarker GWAS summary statistics for prioritized pathway nodes.

Candidate metabolites for TM and MO integration were selected from the pathway-guided stages described above, with particular emphasis on nodes that were mechanistically proximal to the highlighted glycolysis/lactate axis or otherwise retained informative signals in UVMR/MVMR. Triplets were prioritized when TO, TM, and MO evidence showed directional coherence and when the relevant edges were supported by statistical evidence under the corresponding analysis framework. Where available, alternative instrument definitions were examined to assess whether the direction of TM and TO effects was robust to IV specification. This integration step was used to bridge target-level perturbation and pathway-level metabolic interpretation, and key prioritized triangles were summarized in Figure 6 and Table 2.

### 4.12. Summary-Data Two-Step MR Mediation and Paired MVMR Robustness Analysis

To examine whether selected target–outcome associations might operate through downstream clinical or metabolic intermediates, we employed a two-step summary-data MR framework for mediation analysis [42], with paired target + mediator MVMR used as a robustness analysis [50]. Step A estimated target-to-mediator effects using the same cis-target proxy definitions as Section 4.9, with BMI, WHR, fasting insulin, and HOMA-B treated as summary-data outcomes. Step B estimated mediator-to-OC effects using mediator instruments and summary-level OC outcomes. Thus, the inputs in Section 4.12 were harmonized SNP–target-proxy, SNP–mediator, and SNP–OC coefficients extracted from public GWAS datasets, not raw participant-level observations. The participant-level source-study models used to generate the principal OC summary statistics (e.g., OCAC logistic regression and UK Biobank BOLT-LMM) are summarized in Supplementary Table S1 rather than being newly analyzed here.

Effect estimation in Steps A and B followed the same summary-data MR framework used elsewhere in the manuscript (IVW for multi-SNP instrument sets and the Wald ratio for single-SNP analyses), implemented in R using ieugwasr and TwoSampleMR [21,38]. Harmonization and proxy handling followed the rules described in Section 4.3, and threshold/proxy substitutions used in the mediation analyses are documented in Supplementary Table S11. The indirect effect was calculated on the log scale as β_indirect_ = β_A_ × β_B_; its standard error was estimated by the delta method, and the mediated proportion was computed as β_indirect_/β_total_, where β_total_ was the corresponding univariable target → outcome estimate on the log scale [56]. For presentation of binary OC outcomes, indirect effects were exponentiated to the OR scale.

Because product-of-coefficients mediation can be sensitive to correlated instruments and mediator specification, we additionally fitted paired target + mediator MVMR models as a robustness layer to compare the total target effect with the direct effect after mutual adjustment [50,56]. This paired MVMR step was not a new discovery screen; rather, it was used to assess whether the target → outcome estimate materially attenuated after inclusion of the proposed mediator. Full mediation and robustness results are reported in Supplementary Table S11.

### 4.13. Multiplicity Strategy, Sample Overlap, Ancestry Considerations, and Software

This study combined exploratory nomination stages and hypothesis-guided follow-up stages; accordingly, multiple testing was handled within predefined analysis families rather than by a single global correction across the entire workflow. All raw *p* values are reported, but the inferential status of an association depends on the analytical stage in which it arose.

At the metabolome-wide screening stage (Section 4.5), IVW *p* < 0.05 was used only as a nomination threshold to carry forward candidate metabolites for pathway summarization and downstream biological follow-up. These metabolite-level screening results were therefore interpreted as hypothesis-generating rather than confirmatory. KEGG pathway enrichment of the IVW-positive metabolite sets was then performed separately for each overall OC outcome, and multiplicity within each enrichment run was controlled using the Benjamini–Hochberg false discovery rate (FDR) procedure [24]. Accordingly, both raw *p* values and FDR-adjusted significance were considered in pathway interpretation (Supplementary Table S4).

For pathway-guided histotype-stratified UVMR and BWMR follow-up (Section 4.6 and Section 4.7), the primary aim was biological tracing and prioritization along the prespecified amino acid/energy–nitrogen axis. Unless otherwise stated, these results were treated as exploratory and are reported mainly with nominal *p* values, while BWMR served as a robustness estimator rather than as a separate multiplicity-controlled discovery analysis.

For pathway-restricted MVMR (Section 4.8), multiplicity was controlled within each prespecified primary model family using the Benjamini–Hochberg FDR procedure across the tested OC outcomes, and *q* values are reported in Supplementary Table S6. For drug-target MR (Section 4.9), multiplicity was controlled within each target across OC outcomes/histotypes in the primary merged cis-IV analysis using Benjamini–Hochberg FDR; FDR-significant findings are indicated in Figure 5A and detailed in Supplementary Table S7. Alternative IV definitions, strict-clumping analyses, and LD-aware correlated-SNP models were interpreted as sensitivity analyses for robustness rather than as separate discovery families.

Section 4.11 and Section 4.12 (triangular evidence integration and two-step mediation/MVMR robustness) were used for mechanistic triangulation after target and pathway prioritization. These steps were not intended as independent high-dimensional discovery stages and were interpreted primarily on the basis of directional consistency, attenuation patterns, and biological coherence, with nominal *p* values reported for transparency.

Because some metabolite GWAS may include UK Biobank participants, partial sample overlap with the UK Biobank OC outcome is possible. Under weak instruments, overlap may bias estimates towards observational associations [40]; therefore, we used OCAC outcomes as an independent comparison dataset and reported instrument-strength diagnostics to mitigate overlap-related bias.

Some exposure GWASs (e.g., lactate dehydrogenase from Biobank Japan (Tokyo, Japan)) are non-European, whereas most OC outcomes are European. Cross-ancestry MR may be affected by LD and allele-frequency differences; such findings were interpreted cautiously and supported by sensitivity and consistency evidence.

Analyses were performed in R (version 4.5.2; R Foundation for Statistical Computing, Vienna, Austria; https://www.r-project.org/, accessed on 29 May 2026) on macOS Sonoma 14.4.1 (aarch64-apple-darwin20). Key package versions were: TwoSampleMR 0.6.29, ieugwasr 1.1.0, MendelianRandomization 0.10.0, MR-PRESSO 1.0, coloc 5.2.3, data.table 1.18.0, and dplyr 1.1.4.

### 4.14. Experimental Validation in Ovarian Cancer Cell Models (Newly Generated Laboratory Data)

Unlike Section 4.2, Section 4.3, Section 4.4, Section 4.5, Section 4.6, Section 4.7, Section 4.8, Section 4.9, Section 4.10, Section 4.11 and Section 4.12, which describe the re-analysis of publicly available GWAS/eQTL summary statistics, Section 4.14, Section 4.15 and Section 4.16 describe newly generated cell-based experiments performed by the authors. These wet-lab experiments were designed to biologically interrogate the MR-prioritized *PPARG/ABCC8/KCNJ11*–lactate–IMOC triangle rather than to generate additional omics datasets. RMUG-S was used as the primary mucinous ovarian cancer model [25]. MCAS was used as an additional mucinous model for supplementary pharmacological validation [27], and OVCAR8 was used as a non-mucinous comparator [28]. *PPARG* perturbation was examined by siRNA-mediated knockdown and pharmacological activation with pioglitazone [26]. In the *PPARG* arm, exogenous sodium lactate supplementation was used as a metabolic rescue condition. *ABCC8/KCNJ11* signaling was examined by transient *ABCC8* overexpression and by pharmacological modulation of the KATP complex using diazoxide and glibenclamide [29,30]. Unless otherwise specified, the mechanistic rescue experiments shown in Figure 7 and Figure 8 were performed in RMUG-S cells, whereas Supplementary Figure S20 reports additional pioglitazone validation in MCAS and OVCAR8 cells.

For the genetic perturbation experiments, *PPARG*-specific siRNAs and the overexpression plasmid pcDNA3.1-ABCC8-EGFP were introduced by transient transfection using Lipofectamine™ 2000 Transfection Reagent (Thermo Fisher Scientific, 11668019, Waltham, MA, USA). Cells were seeded into 6-well plates 1 day before transfection to reach approximately 40–70% confluence at the time of transfection. On the day of transfection, siRNA or plasmid DNA and Lipofectamine™ 2000 were diluted separately in serum-free Opti-MEM, combined, and incubated at room temperature for 15–20 min to allow formation of transfection complexes. The final siRNA concentration was 50 nM, and 2.0 μg/well plasmid DNA was used for overexpression. Complexes were then added to cells maintained in antibiotic-free complete medium. After 4–6 h, the medium was replaced with fresh complete medium containing 10% FBS. Cells were collected approximately 48 h after transfection. RT-qPCR was used to assess perturbation efficiency at the mRNA level, and immunoblotting was used to validate protein-level perturbation.

### 4.15. Lactate Quantification After Genetic or Pharmacological Perturbation

Extracellular and intracellular lactate were measured in newly generated cell-based samples using the Elabscience L-Lactic Acid (LA) Colorimetric Assay Kit (Elabscience, E-BC-K044-M, Wuhan, China) according to the manufacturer’s instructions. After 72 h of the indicated genetic or pharmacological perturbation, culture supernatants were collected for extracellular lactate measurement, and cells were harvested for intracellular lactate determination. Before intracellular lactate measurement, cells were washed three times with ice-cold PBS, homogenized in PBS, and centrifuged at 10,000× *g* for 10 min at 4 °C. The supernatant was then collected for analysis. Samples were diluted as needed to ensure that the absorbance values fell within the linear range of the standard curve.

For the colorimetric assay, 5 μL of standard or sample was added to each well of a 96-well plate, followed by 100 μL of enzyme working solution and 20 μL of chromogenic reagent. After incubation at 37 °C for 10 min, 180 μL of stop solution was added, and absorbance was measured at 530 nm using a PerkinElmer EnSight multimode plate reader (PerkinElmer, Waltham, MA, USA). Standard curves were generated according to the manufacturer’s instructions. Extracellular lactate concentrations were expressed as mmol/L, whereas intracellular lactate was normalized to total protein and expressed as mmol/gprot. This assay was used for the RMUG-S mechanistic experiments shown in Figure 7 and Figure 8 and for the supplementary pioglitazone validation in MCAS and OVCAR8 cells.

### 4.16. In Vitro Phenotypic Assays and Statistical Analysis of Laboratory Experiments

The following subsections describe the in vitro phenotypic assays performed after the indicated genetic or pharmacological perturbations. Unless otherwise specified, the core experiments were carried out in RMUG-S cells, whereas additional pioglitazone validation in MCAS and OVCAR8 is reported in Supplementary Figure S20.

#### 4.16.1. Colony Formation Assay

Colony formation assays were performed to evaluate long-term proliferative capacity. Cells were seeded into 6-well plates at a density of 2000 cells per well and cultured for 10 days under the indicated treatment conditions, with regular medium replacement during the incubation period. At the endpoint, the medium was discarded, cells were gently washed with PBS, fixed with fixative solution (Servicebio, G1101, Wuhan, China) for 15–30 min, and stained with crystal violet solution (Servicebio, G1014, Wuhan, China) for 10–20 min. Excess dye was removed by washing, and plates were air-dried before imaging. Colony-forming ability was quantified as crystal violet-positive area fraction (%). Each group included three technical replicate wells, and at least three independent experiments were performed.

#### 4.16.2. CCK-8 Cell Viability Assay

Cell viability after pioglitazone treatment was assessed using the SuperKine™ Cell Counting Kit-8 (CCK-8) (Abbkine, BMU106-CN, Wuhan, China). This assay was used primarily to define the RMUG-S working concentration/time condition for the subsequent pioglitazone experiments. Cells were seeded into 96-well plates at a density of 2000 cells per well. After attachment, cells were treated with pioglitazone at final concentrations of 0, 0.3, 1, 3, 10, 30, 80, and 160 μM. Vehicle wells received the same final solvent concentration. After 24, 48, or 72 h of treatment, CCK-8 reagent was added to each well at 10% (*v*/*v*) of the culture medium volume and incubated at 37 °C in 5% CO_2_ for 1–2 h in the dark. Absorbance was then measured at 450 nm using a PerkinElmer EnSight multimode plate reader. Blank wells and vehicle-control wells were included in each experiment. Cell viability was calculated as: Cell viability (%) = [(A_sample − A_blank)/(A_control − A_blank)] × 100, where A_sample, A_control and A_blank represent the absorbance of drug-treated wells, vehicle control wells, and blank wells, respectively.

Each concentration and time point was tested in three technical replicates, and at least three independent experiments were performed. Dose–response curves were generated after normalization to the vehicle group.

#### 4.16.3. Transwell Invasion Assay

Cell invasive capacity was assessed using Transwell inserts (Corning, 3422, New York, NY, USA). Matrigel (Corning, 356234, New York, NY, USA) was diluted 1:9, coated onto the upper membrane surface, and incubated at 37 °C for 1 h to allow gel formation. Cells were then resuspended in serum-free medium and seeded into the upper chamber at 50,000 cells per well, while complete medium containing 10% FBS was added to the lower chamber as a chemoattractant. After 48 h of incubation, non-invading cells on the upper surface were gently removed. Cells that had invaded the lower surface were fixed with fixative solution, stained with crystal violet solution, washed, air-dried, and imaged using an inverted microscope (Olympus IX71, Tokyo, Japan, 200×). Five random fields were selected for counting in each insert, and results were expressed as the number of invading cells per field. Each group included three independent biological replicates.

#### 4.16.4. Wound-Healing Migration Assay

Cells were seeded into 12-well plates at a density of 5 × 10^5^ cells/well and cultured until a confluent monolayer had formed. A linear wound was then generated in the center of each well using a sterile 200 μL pipette tip, and detached cells were gently removed by washing with PBS. The medium was then replaced with low-serum medium containing the indicated treatments. Cell migration was continuously monitored for 48 h using the Incucyte^®^ SX5 live-cell imaging system (Sartorius, Göttingen, Germany), with images automatically acquired every 2 h. Wound areas were identified and quantified using Incucyte 2022B software, and migratory capacity was evaluated as wound closure (%).

#### 4.16.5. Real-Time Cytotoxicity Assay

Cells were seeded into 96-well plates at a density of 4 × 10^3^ cells/well. After attachment, cultures were washed, and the medium was replaced before imaging. The indicated treatments were then applied, and Incucyte^®^ Cytotox Red Dye (Sartorius, 4632, Göttingen, Germany) was added immediately at a final concentration of 250 nM. The dye was maintained in the medium throughout the entire detection period. Cytotoxicity was continuously monitored for 72 h using the Incucyte^®^ SX5 live-cell imaging system (Sartorius, Göttingen, Germany), with phase-contrast and red-fluorescence images automatically acquired every 2 h. Images were analyzed using Incucyte 2022B software. Cell death was quantified as Red Area/Phase Area and normalized to the 0 d 0 h 0 min time point.

#### 4.16.6. Data Presentation and Statistical Analysis of Laboratory Experiments

Unless otherwise specified, laboratory data are presented as mean ± SD from three independent experiments. Colony formation and CCK-8 assays included three technical replicate wells per condition within each experiment, whereas Transwell assays were performed with three independent biological replicates. For wound-healing and real-time cytotoxicity assays, images were acquired longitudinally and quantified using Incucyte 2022B software. Two-group comparisons were analyzed using a two-tailed Student’s *t* test, and comparisons among three or more groups were analyzed using one-way ANOVA. The statistical tests used for the individual experiments are also indicated in the corresponding figure legends.

## 5. Conclusions

In summary, by integrating histotype-stratified metabolome-wide MR, pathway tracing, pathway-restricted MVMR, drug-target MR, mediation analysis, and experimental interrogation, this study establishes a histotype-centered genetic causal framework for ovarian cancer. Apparently, dispersed metabolomic signals are thereby reorganized into an interpretable mechanistic axis spanning amino acids, the TCA cycle, glycolysis/lactate-end states, and urea-cycle/polyamine metabolism, while prioritized target–metabolite–histotype relationships are further supported by a lactate-centered triangular chain of evidence. Overall, this framework moves beyond single-metabolite association signals by combining mechanistic interpretability, target mappability, and experimental testability, and provides a clearer roadmap for the stepwise validation of prevention and intervention hypotheses across cohorts, pharmacoepidemiology, and experimental systems.

## Figures and Tables

**Table 1:** Summary of external datasets used in the present study, including data level and analytical role.

Analysis Component	Dataset/Trait(s)	Data Level Used in the Present Study	Source/OpenGWAS ID(s)	Sample Size (*N*; Cases/Controls if Applicable)	Ancestry	Analytical Role in the Present Study
Metabolome-wide screening exposures	1400 plasma metabolites/ratios	External GWAS summary statistics only; no individual-level data used	Chen et al.; ebi-a-GCST90199621–ebi-a-GCST90201020 (full list in Supplementary Table S2)	*N* = 3441–8299 per trait	European	Metabolome-wide MR screening against overall ovarian cancer
Follow-up pathway exposures	Selected amino acids, TCA intermediates, glycolysis/lactate traits, urea/polyamine nodes, and enzyme/protein proxies	External GWAS/pQTL summary statistics only	met-a/met-c/met-d/selected ebi-a/prot-a series; bbj-a-30 for LDH; full trait-level IDs in Supplementary Table S1	Trait-specific	Mostly European; LDH biomarker from East Asian GWAS	Pathway-tracing UVMR and pathway-restricted MVMR
Drug-target instrument source	Blood-glucose GWAS used for online cis-IV extraction	External GWAS summary statistics only	ebi-a-GCST90025986	Up to 400,458	European	Construction of online cis instruments for drug-target MR
Drug-target analyses	Glucose-lowering drug-target proxies (full initial target panel listed in Supplementary Table S1; prioritized main-text targets include *PPARG*, *DPP4*, *ABCC8/KCNJ11*, and *SLC5A2*)	External summary statistics only; cis-SNP instrument sets	Published cis-instrument sources plus online cis-IVs derived from the blood-glucose GWAS; full target/SNP details in Supplementary Tables S7 and S10	Not represented by a single GWAS ID	Mainly European	Drug-target MR, sensitivity analyses, and target–metabolite triangulation
Mediators	BMI; WHR; fasting insulin; HOMA-B	External GWAS summary statistics only	ukb-b-19953; ieu-a-81; ieu-b-116; ieu-b-117	*N* = 36,466–461,460	European	Two-step MR mediation and MVMR robustness analyses
Outcomes (overall)	Overall ovarian cancer (OCAC)	External GWAS summary statistics only	ieu-a-1120	*N* = 66,450; 25,509/40,941	European	Independent overall ovarian cancer outcome used in screening and downstream comparison
Outcomes (overall)	Overall ovarian cancer (UK Biobank)	External GWAS summary statistics only	ieu-b-4963	*N* = 199,741; 1218/198,523	European	Independent overall ovarian cancer outcome used in screening/replication
Outcomes (histotype/LMP)	OCAC histotype-specific and low-malignant-potential outcomes	External GWAS summary statistics only	ieu-a-1121 to ieu-a-1125; ieu-a-1228 to ieu-a-1233	Trait-specific; see Supplementary Table S1	European	Histotype-stratified UVMR/MVMR, drug-target MR, triangulation, and mediation
Regulatory datasets	cis-eQTL datasets for colocalization	External cis-eQTL summary statistics only	eQTLGen whole blood; GTEx v8 tissues	Tissue-/dataset-specific; see Supplementary Table S1	Source-specific	Two-stage cis colocalization

GWAS sources are summarized by data platform for exposures, mediators/covariates, and outcomes, including OpenGWAS IDs (or cis-instrument set labels), sample size, ancestry, and study usage. Complete dataset metadata and MR parameter settings are provided in Supplementary Table S1; the full list of 1400 metabolites is provided in Supplementary Table S2.

**Table 2:** Key target–mediator–outcome chains (key pairs): summary of two-step MR mediation and MVMR robustness analysis.

Target	Mediator	OC Outcome	Total Effect (Univariable)	Direct Effect (MVMR Adj.)	Attenuation	Indirect Effect (Two-Step)	Mediated (%)
*ABCC8/KCNJ11*	BMI	Serous LMP	0.22 (0.08–0.59); *p* = 0.003	0.12 (0.04–0.38); *p* = 3.99 × 10^–4^	0% (no atten.)	0.91 (0.84–0.98); *p* = 0.011	6.5%
*ABCC8/KCNJ11*	BMI	LMP-OC	0.37 (0.16–0.84); *p* = 0.018	0.32 (0.12–0.83); *p* = 0.020	0% (no atten.)	0.93 (0.87–0.98); *p* = 0.013	7.7%
*ABCC8/KCNJ11*	BMI	Serous(LG + LMP)	0.44 (0.19–1.00); *p* = 0.050	0.31 (0.12–0.83); *p* = 0.019	0% (no atten.)	0.94 (0.89–0.99); *p* = 0.030	7.5%
*ABCC8/KCNJ11*	BMI	Overall OC (OCAC)	0.52 (0.34–0.78); *p* = 0.002	0.78 (0.50–1.22); *p* = 0.273	62%	0.97 (0.95–1.00); *p* = 0.039	4.3%
*PPARG*	BMI	Serous LMP	0.17 (0.10–0.27); *p* = 1.19 × 10^–13^	0.12 (0.08–0.20); *p* = 2.69 × 10^–17^	0% (no atten.)	0.97 (0.94–1.00); *p* = 0.041	1.7%
*ABCC8/KCNJ11*	BMI	Endometrioid	0.31 (0.14–0.71); *p* = 0.005	0.59 (0.23–1.55); *p* = 0.283	55%	0.95 (0.90–1.00); *p* = 0.042	4.7%
*PPARG*	BMI	LMP-OC	0.41 (0.28–0.60); *p* = 5.73 × 10^–6^	0.31 (0.21–0.45); *p* = 3.89 × 10^–9^	0% (no atten.)	0.98 (0.95–1.00); *p* = 0.044	2.6%
*DPP4*	HOMA-B	Inv mucinous	0.10 (0.03–0.31); *p* = 4.94 × 10^–5^	0.06 (0.00–0.66); *p* = 0.022	0% (no atten.)	0.46 (0.24–0.91); *p* = 0.025	33.4%

This table summarizes key target–mediator–outcome pairs identified by two-step MR mediation and further evaluated by MVMR to assess attenuation of the target effect after adjustment for the mediator.

## Data Availability

The datasets supporting the conclusions of this article are available in the IEU OpenGWAS repository (MR-Base infrastructure, https://opengwas.io/, accessed on 29 May 2026). All OpenGWAS IDs and original sources for the GWAS summary statistics analyzed in this study are provided in Table 1 and Supplementary Tables S1 and S2, and can be accessed via https://opengwas.io/datasets/<OpenGWAS_ID> (accessed on 29 May 2026) (e.g., ovarian cancer outcomes: https://opengwas.io/datasets/ieu-b-4963[UK Biobank] (accessed on 29 May 2026) and https://opengwas.io/datasets/ieu-a-1120[OCAC] (accessed on 29 May 2026); blood glucose GWAS used for cis-instrument extraction: https://opengwas.io/datasets/ebi-a-GCST90025986 (accessed on 29 May 2026). eQTL summary statistics used for cis colocalization are publicly available from the eQTLGen consortium (https://www.eqtlgen.org/, accessed on 29 May 2026) and the GTEx Portal (v8, https://gtexportal.org/home/, accessed on 29 May 2026). The metabolome-wide screening panel list (*n* = 1400 traits) and other supporting materials (dataset metadata, prespecified analysis settings, step-specific instrument lists, and diagnostic outputs) are included within the article and its Supplementary Information Files (Supplementary Tables S1–S11). No individual-level data were accessed or generated in this study. The analysis scripts used in this study have been deposited in a public GitHub repository: https://github.com/1831259596-stack/ovarian-cancer-histotype-MR-code (accessed on 29 May 2026). Code version: v1.0.0-IJMS-revision. A versioned release corresponding to the revision-stage code set is available at: https://github.com/1831259596-stack/ovarian-cancer-histotype-MR-code/releases/tag/v1.0.0-IJMS-revision (accessed on 29 May 2026).

## Supplementary Materials

The following supporting information can be downloaded at: https://www.mdpi.com/article/10.3390/ijms27115043/s1.

## Abbreviations

αKG, alpha-ketoglutarate (2-oxoglutarate); *ABCC8*, ATP-binding cassette subfamily C member 8; *ABCC8/KCNJ11*, ATP-sensitive potassium (KATP) channel complex genes *ABCC8* and *KCNJ11*; ANOVA, analysis of variance; ASL, argininosuccinate lyase; ASS1, argininosuccinate synthase 1; BBJ, Biobank Japan; BMI, body mass index; BWMR, Bayesian weighted Mendelian randomization; CI, confidence interval; cis-eQTL, cis-expression quantitative trait locus; cis-IV, cis instrumental variable; DAG, directed acyclic graph; DNA, deoxyribonucleic acid; *DPP4*, dipeptidyl peptidase 4; EGFP, enhanced green fluorescent protein; ELBO, evidence lower bound; EM, expectation-maximization; eQTL, expression quantitative trait locus; *ETFDH*, electron transfer flavoprotein dehydrogenase; F, F statistic; FBS, fetal bovine serum; FDR, false discovery rate; GCST, GWAS Catalog study identifier; *GLP1R*, glucagon-like peptide-1 receptor; GLOBOCAN, Global Cancer Observatory; *GPD2*, glycerol-3-phosphate dehydrogenase 2; GRCh37/hg19, Genome Reference Consortium Human Build 37/hg19; GTEx, Genotype-Tissue Expression; GWAS, genome-wide association study; HGSOC, high-grade serous ovarian cancer; HOMA-B, homeostasis model assessment of β-cell function; IEU, Integrative Epidemiology Unit; IMOC, invasive mucinous ovarian cancer; *INSR*, insulin receptor; IV, instrumental variable; IVW, inverse-variance weighted; IVW-MVMR, inverse-variance weighted multivariable Mendelian randomization; KATP, ATP-sensitive potassium channel; *KCNJ11*, potassium inwardly rectifying channel subfamily J member 11; kb, kilobase; KEGG, Kyoto Encyclopedia of Genes and Genomes; LD, linkage disequilibrium; LDH, lactate dehydrogenase; LG, low grade; LG + LMP, low grade plus low malignant potential; LGSOC, low-grade serous ovarian cancer; LMP-OC, low-malignant-potential ovarian cancer; MAF, minor allele frequency; Mb, megabase; MCAS, mucinous ovarian cancer cell line MCAS; MO, metabolite-to-outcome; mRNA, messenger RNA; MR, Mendelian randomization; MR-Base, Mendelian randomization base platform; MR-Egger, MR-Egger regression; MR-PRESSO, Mendelian Randomization Pleiotropy RESidual Sum and Outlier; MVMR, multivariable Mendelian randomization; OC, ovarian cancer; OCAC, Ovarian Cancer Association Consortium; ODC, ornithine decarboxylase; OpenGWAS, IEU OpenGWAS database; OR, odds ratio; OVCAR8, ovarian cancer cell line OVCAR8; PBS, phosphate-buffered saline; PDH, pyruvate dehydrogenase; PP0–PP4, posterior probabilities for colocalization hypotheses H0–H4; PP3, posterior probability for distinct causal variants; PP4, posterior probability for a shared causal variant; PP4_over, PP4/(PP3 + PP4); *PPARG*, peroxisome proliferator-activated receptor gamma; PPARγ, peroxisome proliferator-activated receptor gamma protein; pQTL, protein quantitative trait locus; Q, Cochran’s Q statistic; QC, quality control; r^2^, squared correlation coefficient; RMUG-S, mucinous ovarian cancer cell line RMUG-S; rsID, reference SNP cluster ID; RT-qPCR, real-time quantitative polymerase chain reaction; SD, standard deviation; SE, standard error; SEM, standard error of the mean; siRNA, small interfering RNA; *SLC5A2*, solute carrier family 5 member 2; SNP, single-nucleotide polymorphism; STROBE-MR, Strengthening the Reporting of Observational Studies in Epidemiology Using Mendelian Randomization; TCA, tricarboxylic acid cycle; TM, target-to-metabolite; TO, target-to-outcome; UKB, UK Biobank; UVMR, univariable Mendelian randomization; WHR, waist-to-hip ratio; β-actin, beta-actin.
