# Supplementary material for: From Pathway Tracing to Actionable Targets: Integrative Mendelian Randomization and Experimental Triangulation Map Metabolic Pathways Across Ovarian Cancer Histotypes
Source: Int J Mol Sci. 2026 Jun 2;27(11):5043. doi: 10.3390/ijms27115043 (PMC13256907; doi:10.3390/ijms27115043)
Supplement: Supplementary file 1 [file ijms-27-05043-s001.zip › Supplementary Figures.pdf]

Additional file 2: Supplementary Figures S1–S20.

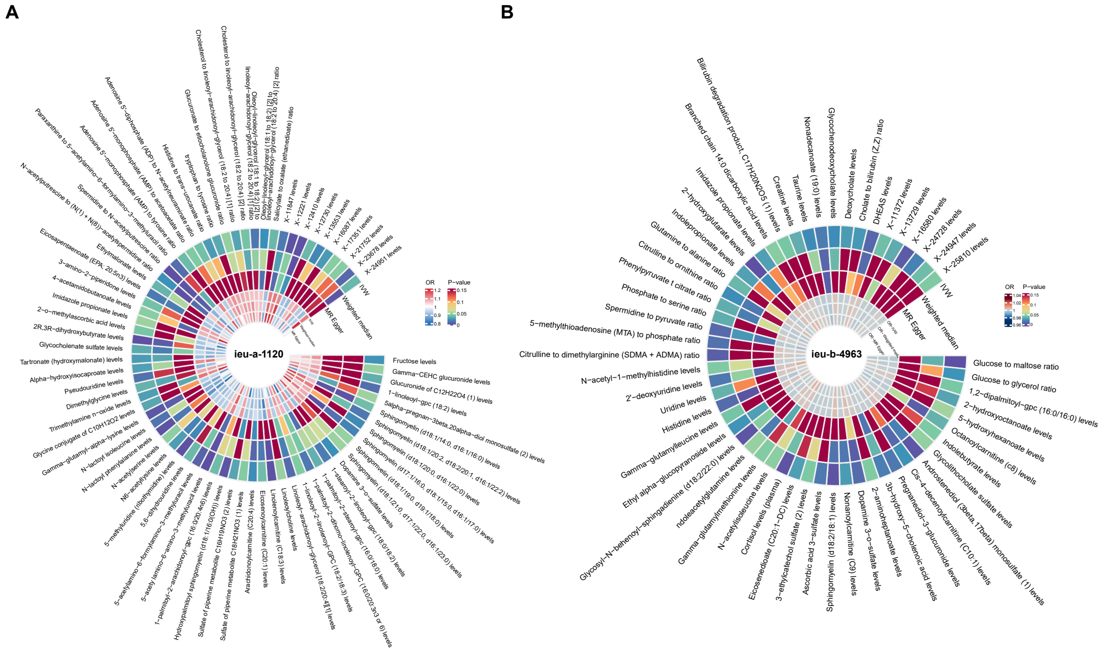

Supplementary Figure S1. Circular heatmaps summarizing TSMR results for candidate metabolites in overall OC.

Candidate metabolites with IVW P<0.05 in (A) ieu-a-1120 (OCAC overall OC) and (B) ieu-b-4963 (UK Biobank overall OC), summarizing P values and ORs across MR methods.

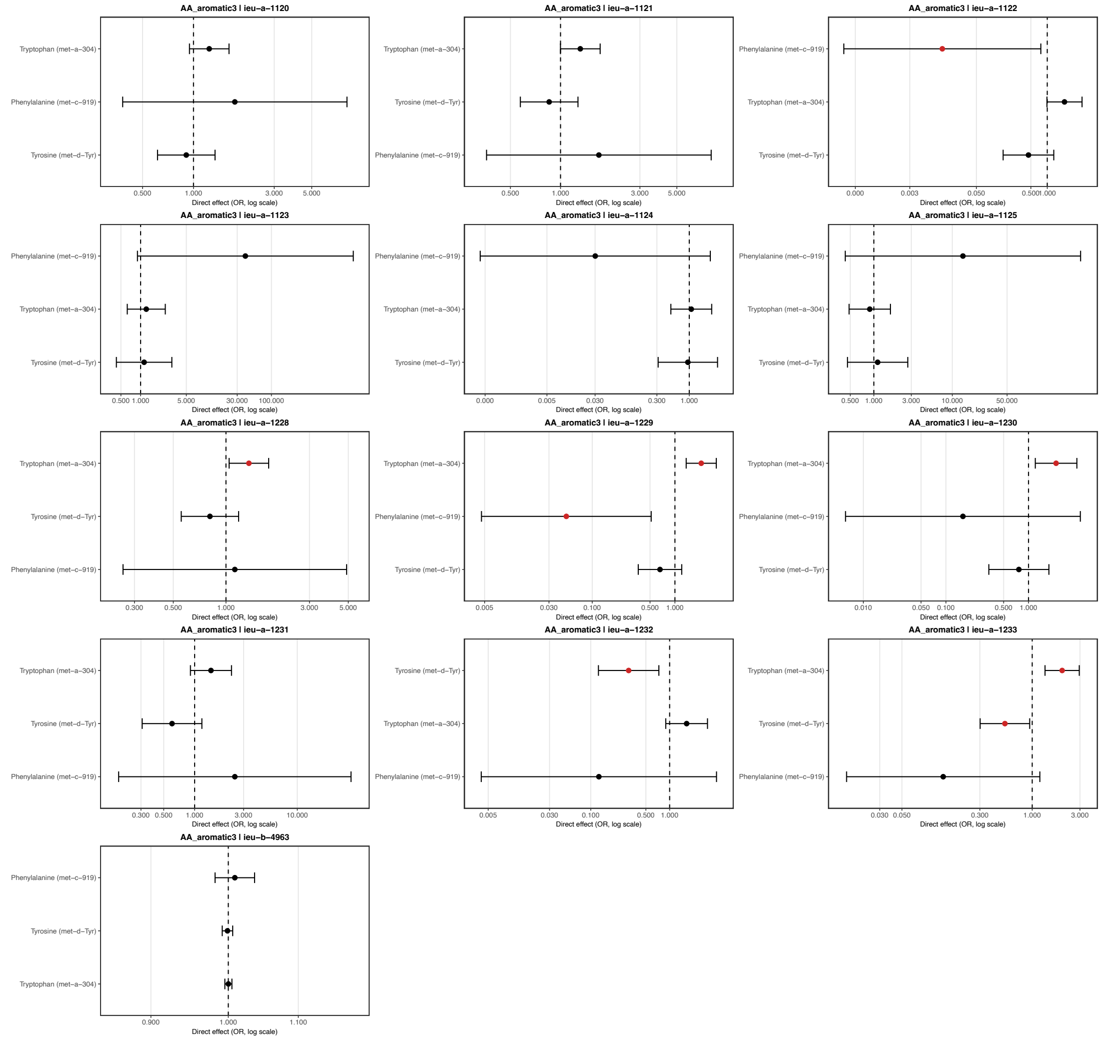

Supplementary Figure S2. Panel B (amino acids): outcome-wide single-page forest plot for the AA_aromatic3 model.

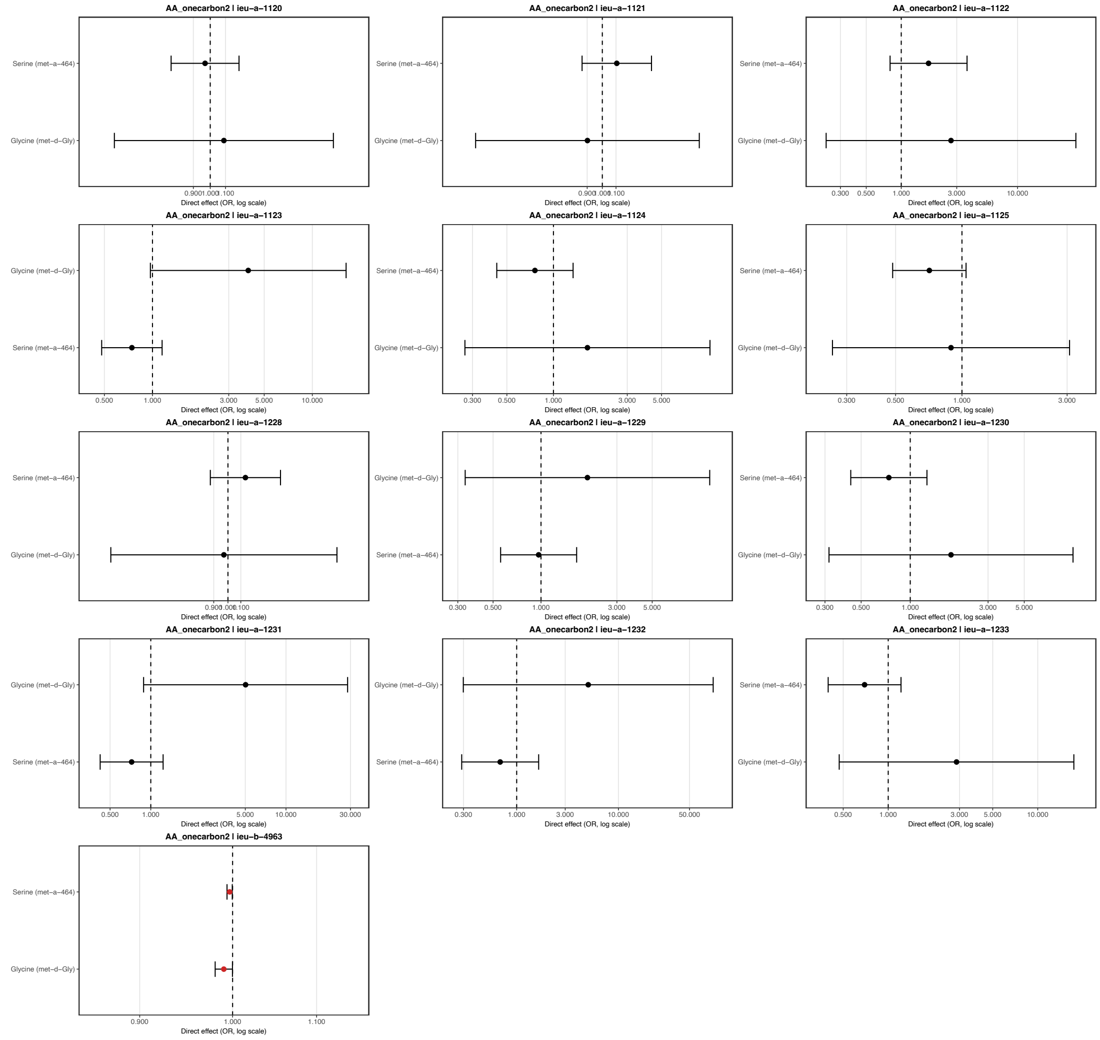

Supplementary Figure S3. Panel B (amino acids): outcome-wide single-page forest plot for the AA_onecarbon2 model.

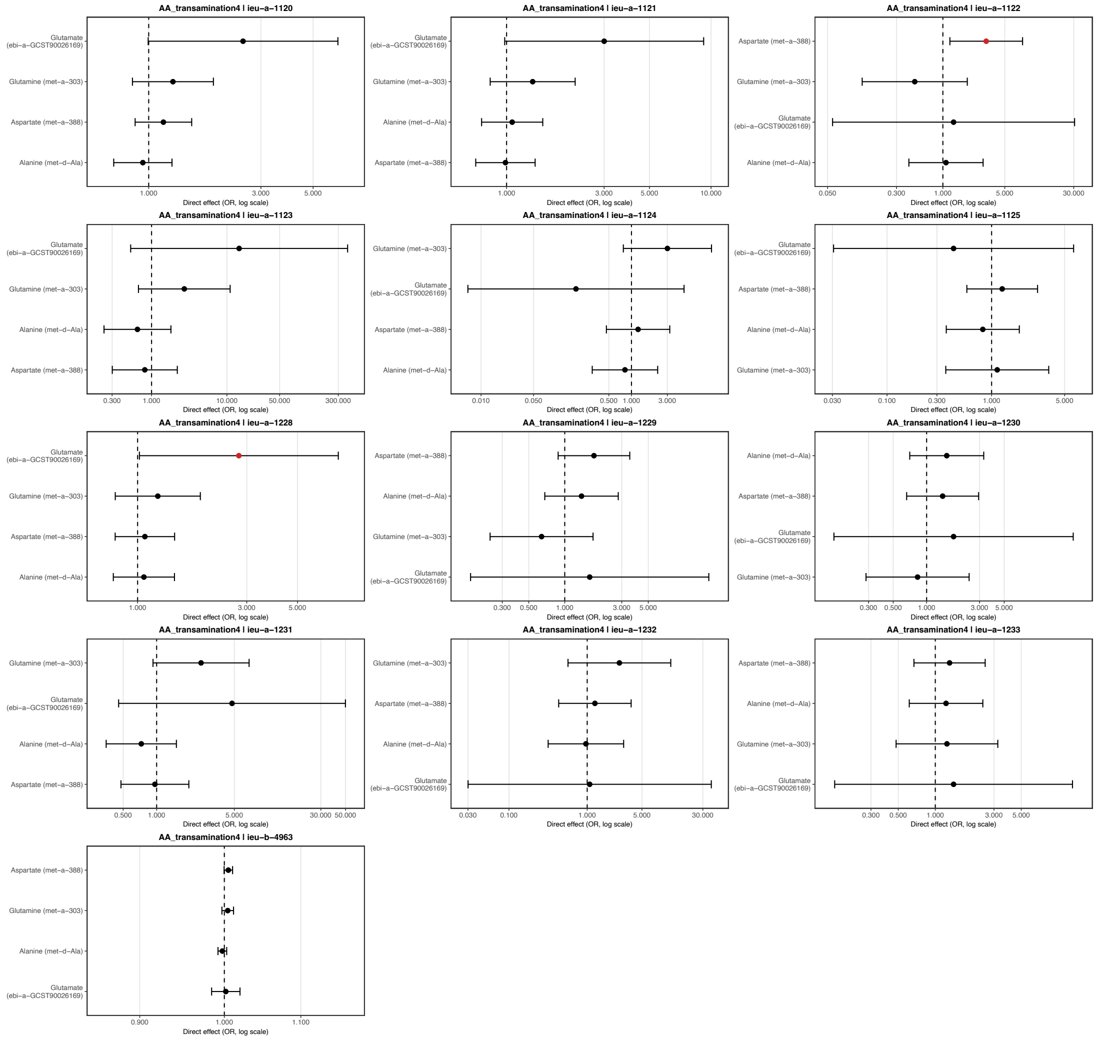

Supplementary Figure S4. Panel B (amino acids): outcome-wide single-page forest plot for the AA_transamination4 model.

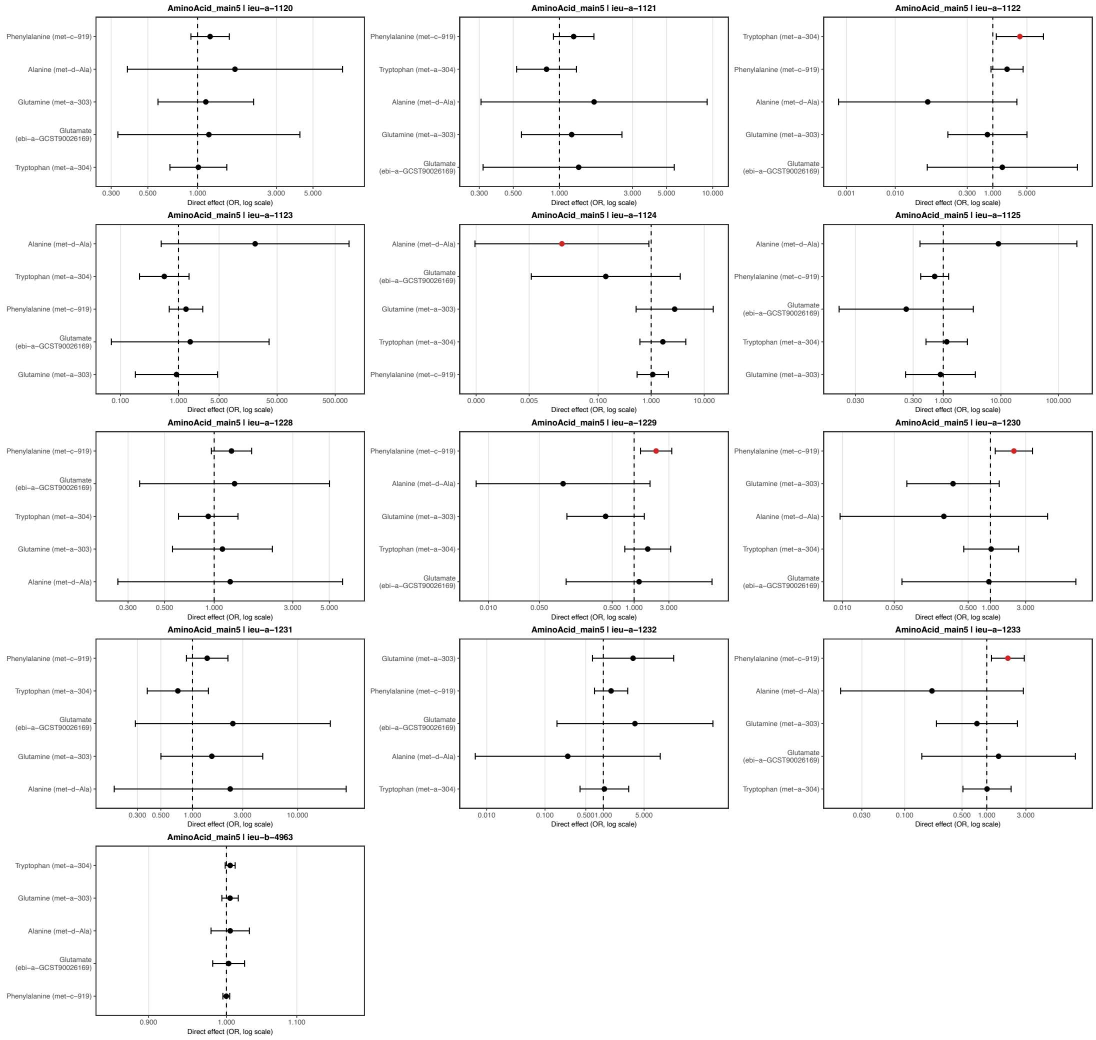

Supplementary Figure S5. Panel B (amino acids): outcome-wide single-page forest plot for the AminoAcid_main5 model.

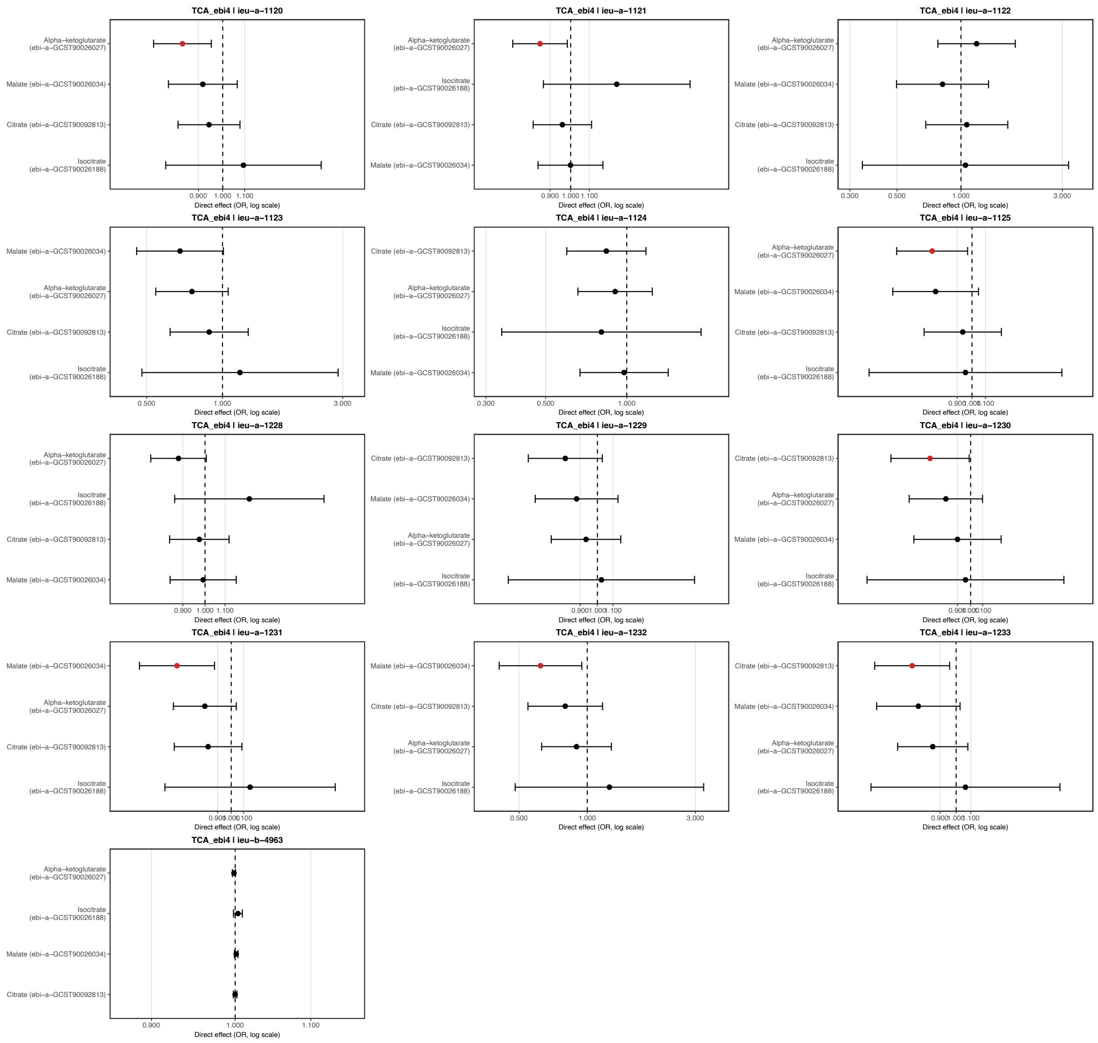

Supplementary Figure S6. Panel C (TCA): outcome-wide single-page forest plot for the TCA_ebi4 model.

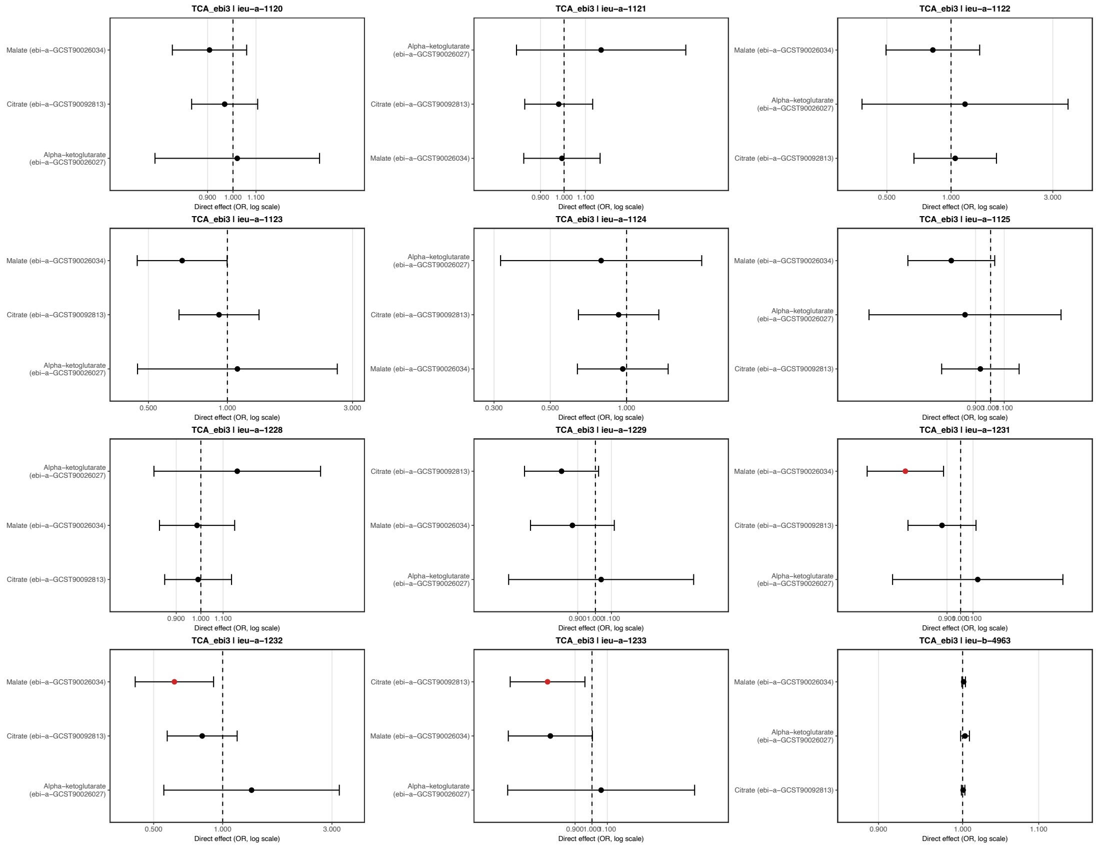

Supplementary Figure S7. Panel C (TCA): outcome-wide single-page forest plot for the TCA_ebi3 dimension-reduced model.

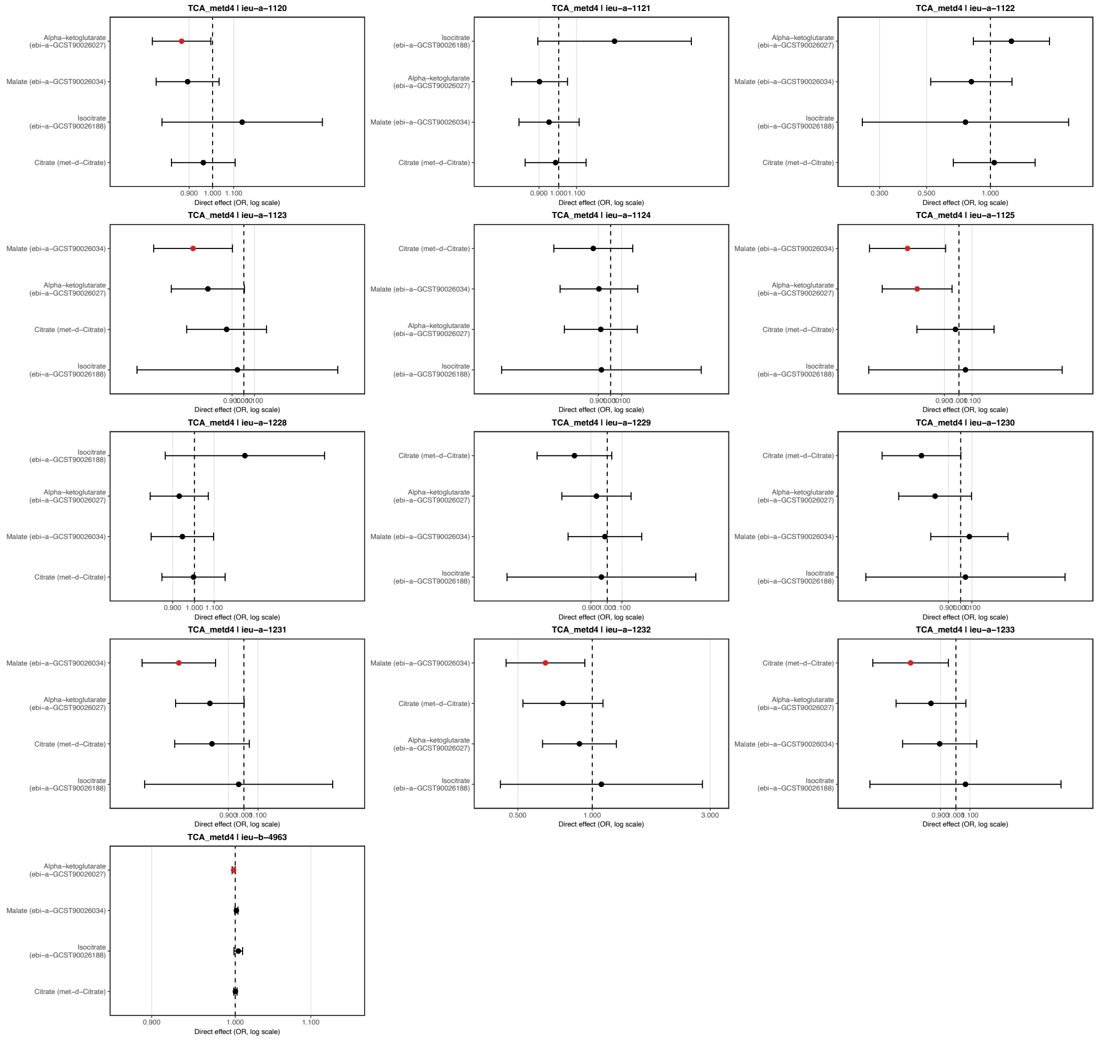

Supplementary Figure S8. Panel C (TCA): outcome-wide single-page forest plot for the TCA_metd4 model.

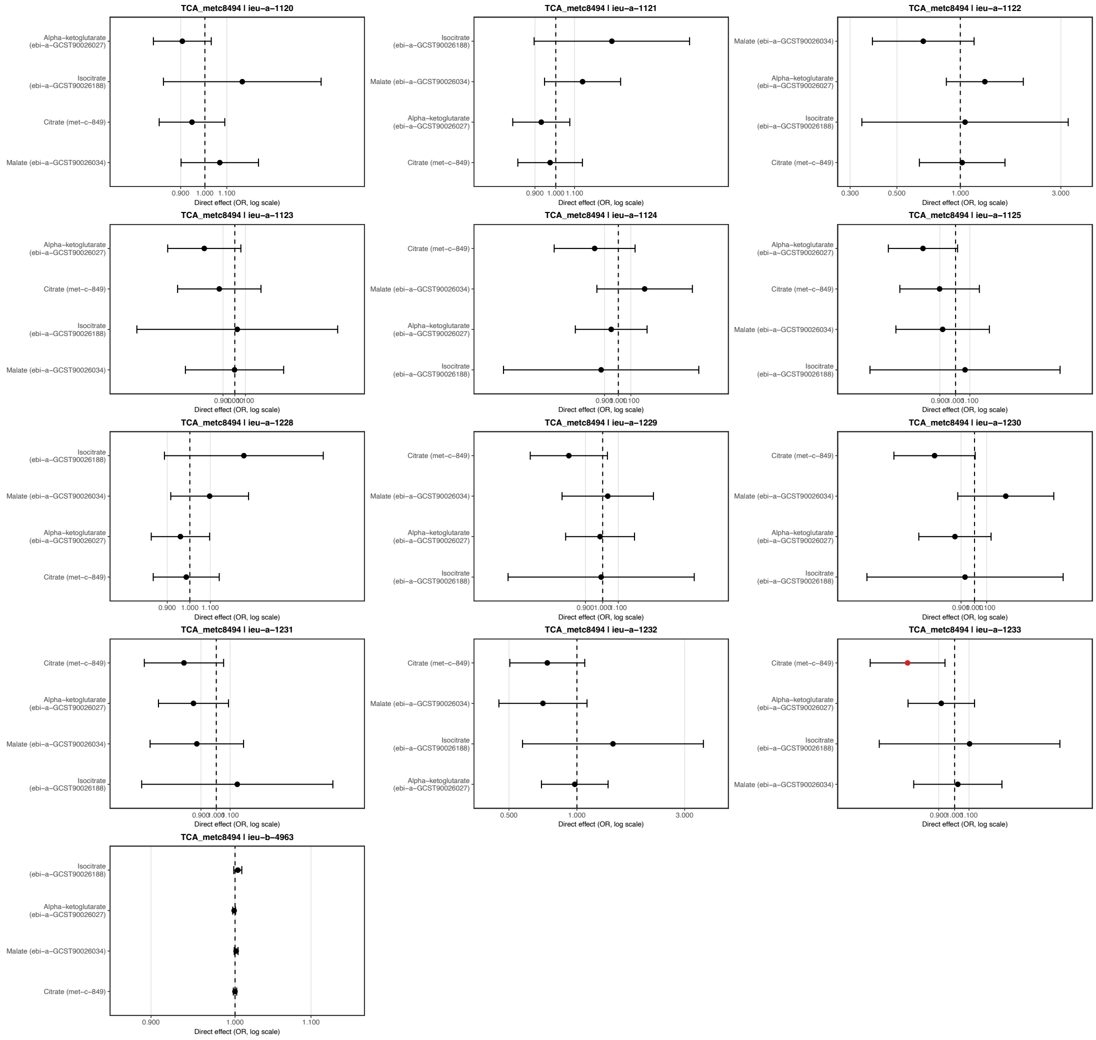

Supplementary Figure S9. Panel C (TCA): outcome-wide single-page forest plot for the TCA_metc8494 model.

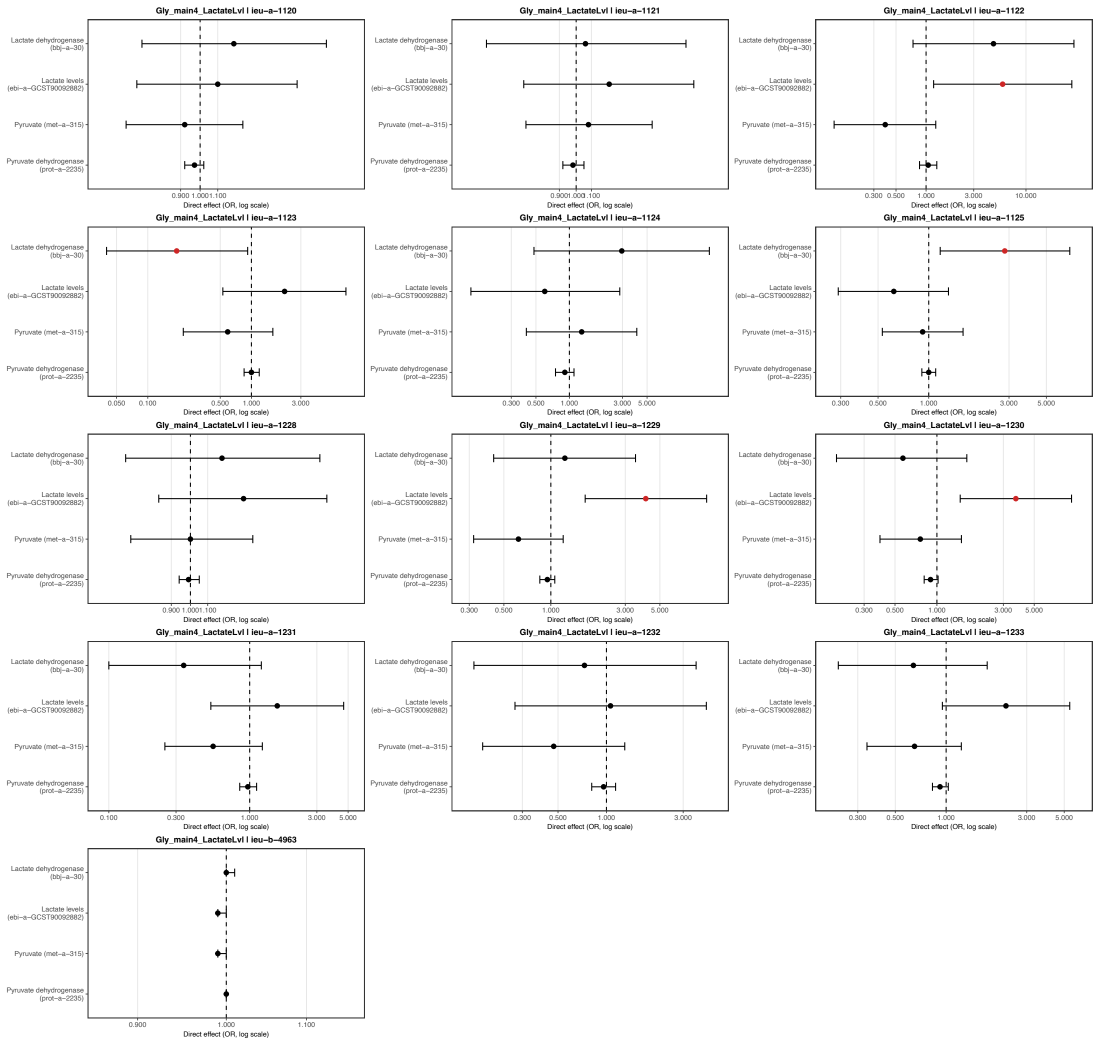

Supplementary Figure S10. Panel D (glycolysis): outcome-wide single-page forest plot for Gly_main4_LactateLvl.

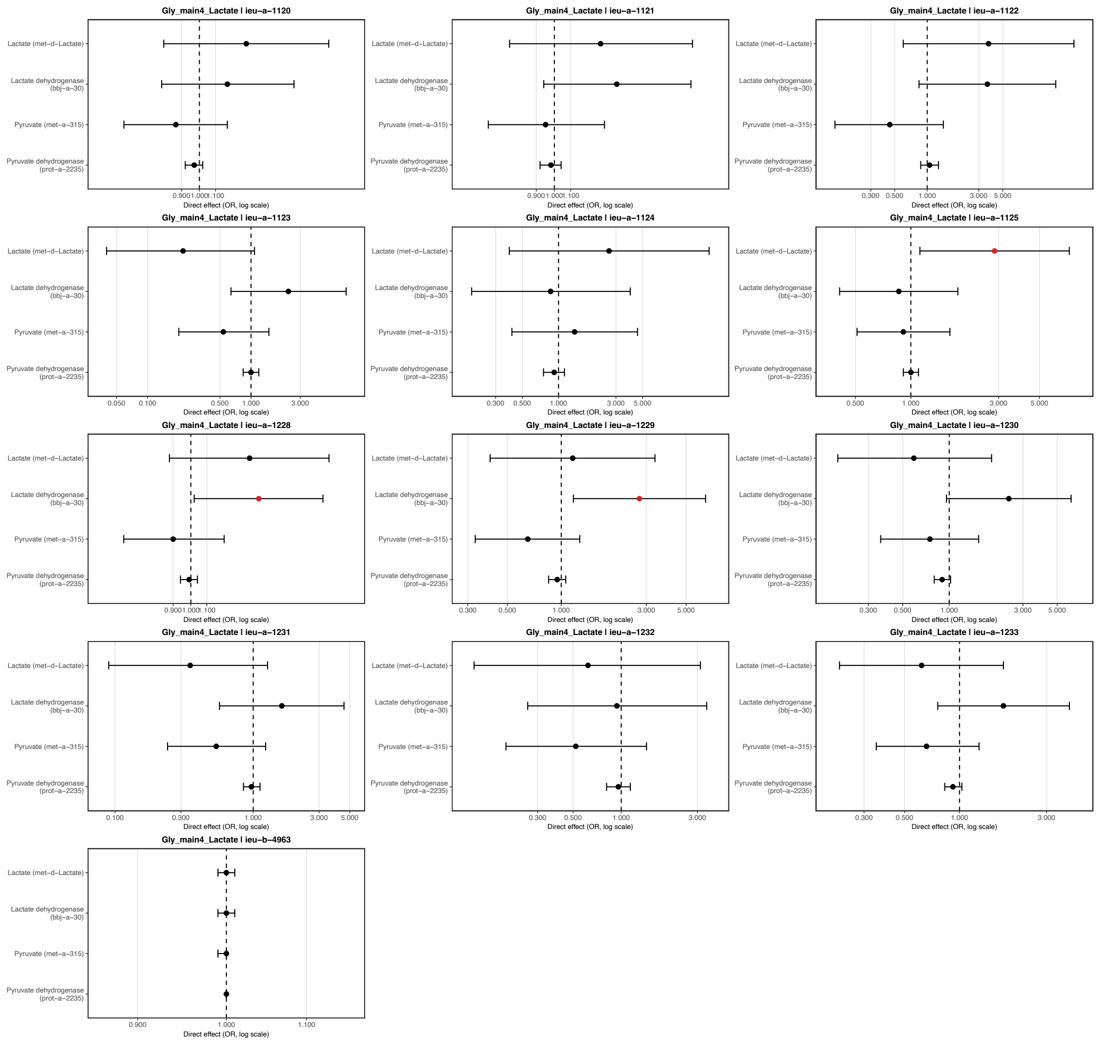

Supplementary Figure S11. Panel D (glycolysis): outcome-wide single-page forest plot for Gly_main4_Lactate (alternative lactate definition).

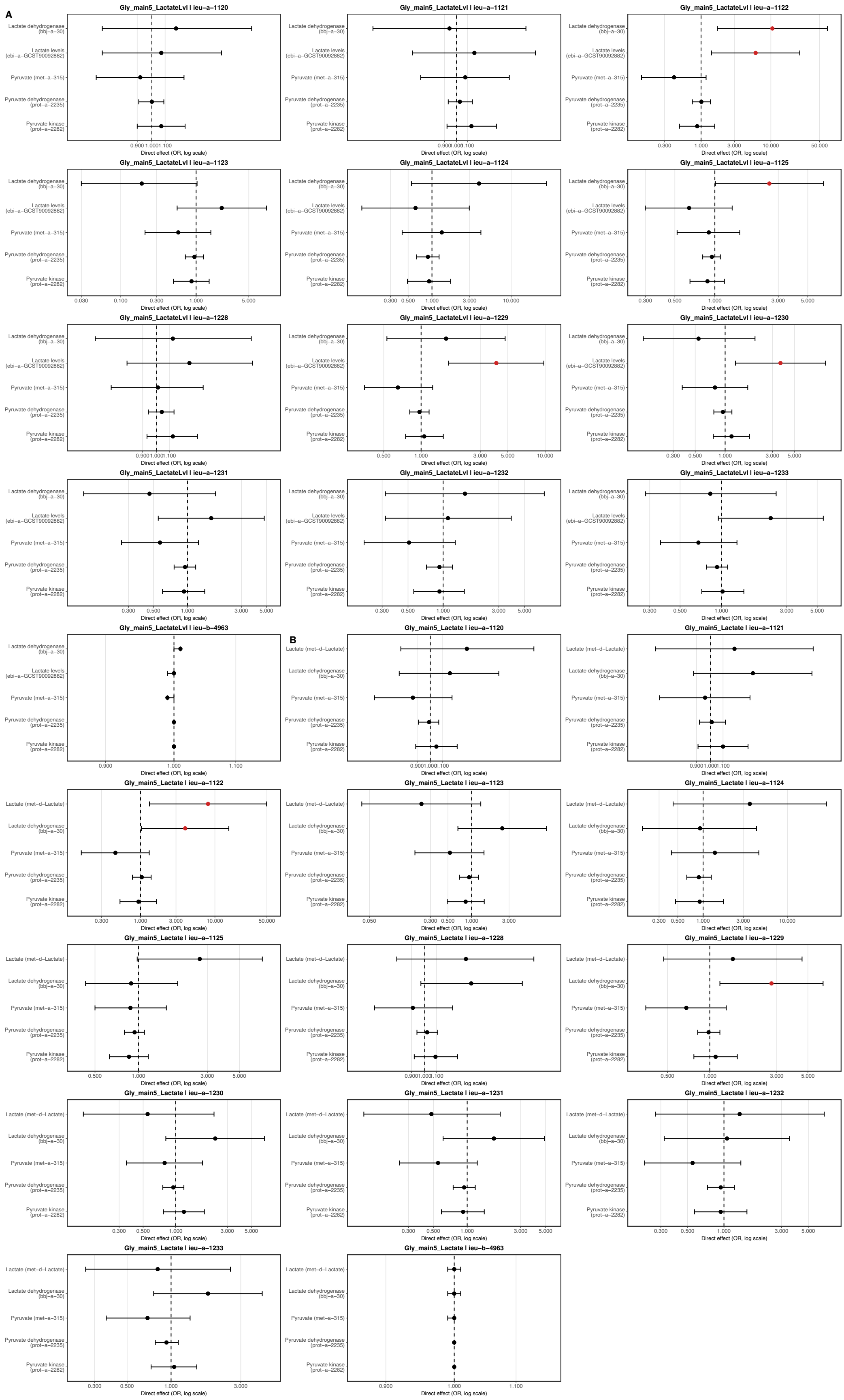

Supplementary Figure S12. Panel D (glycolysis): outcome-wide single-page forest plot for Gly_main5 (including pyruvate kinase).

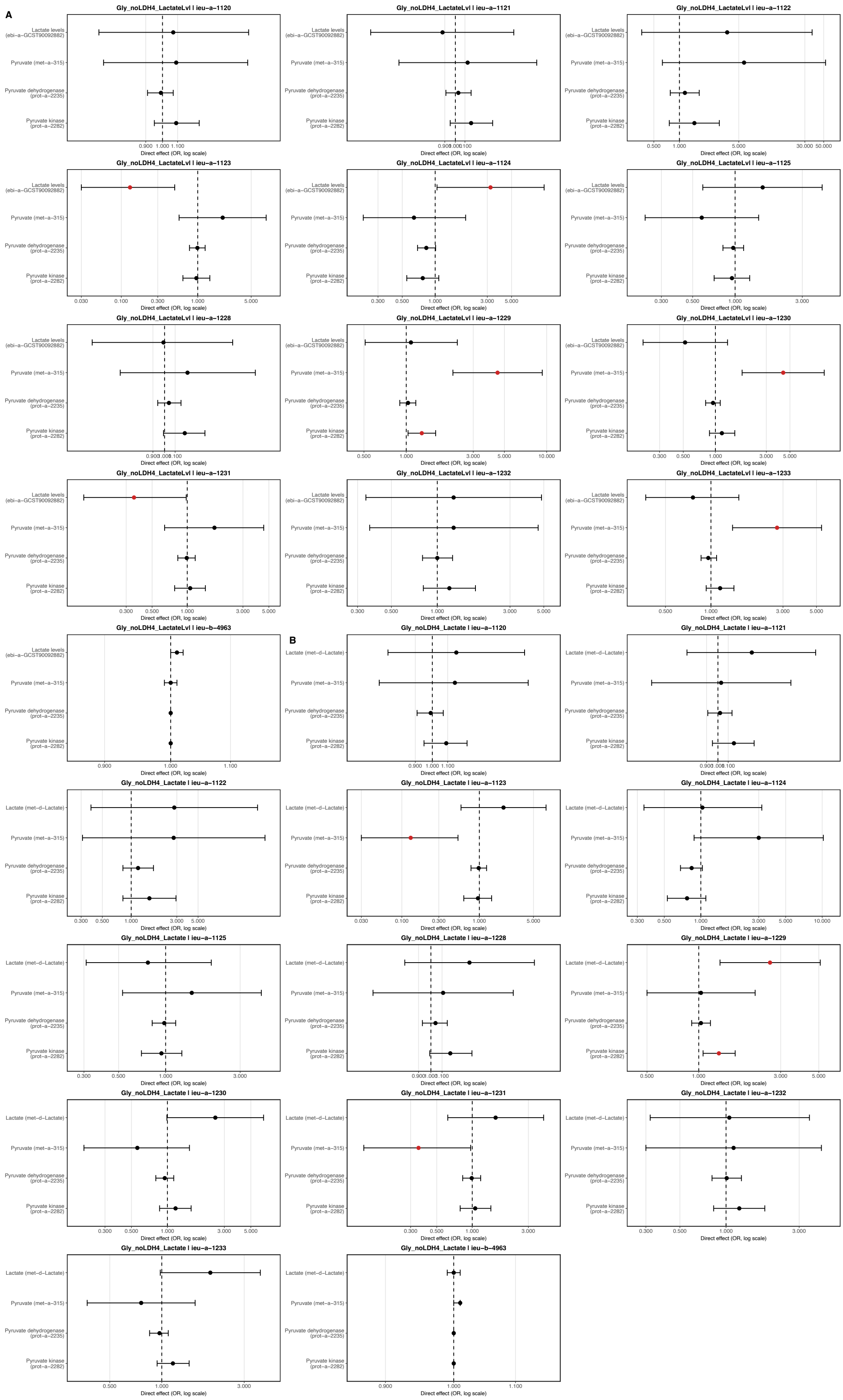

Supplementary Figure S13. Panel D (glycolysis): outcome-wide single-page forest plot for Gly_noLDH4 (excluding LDH).

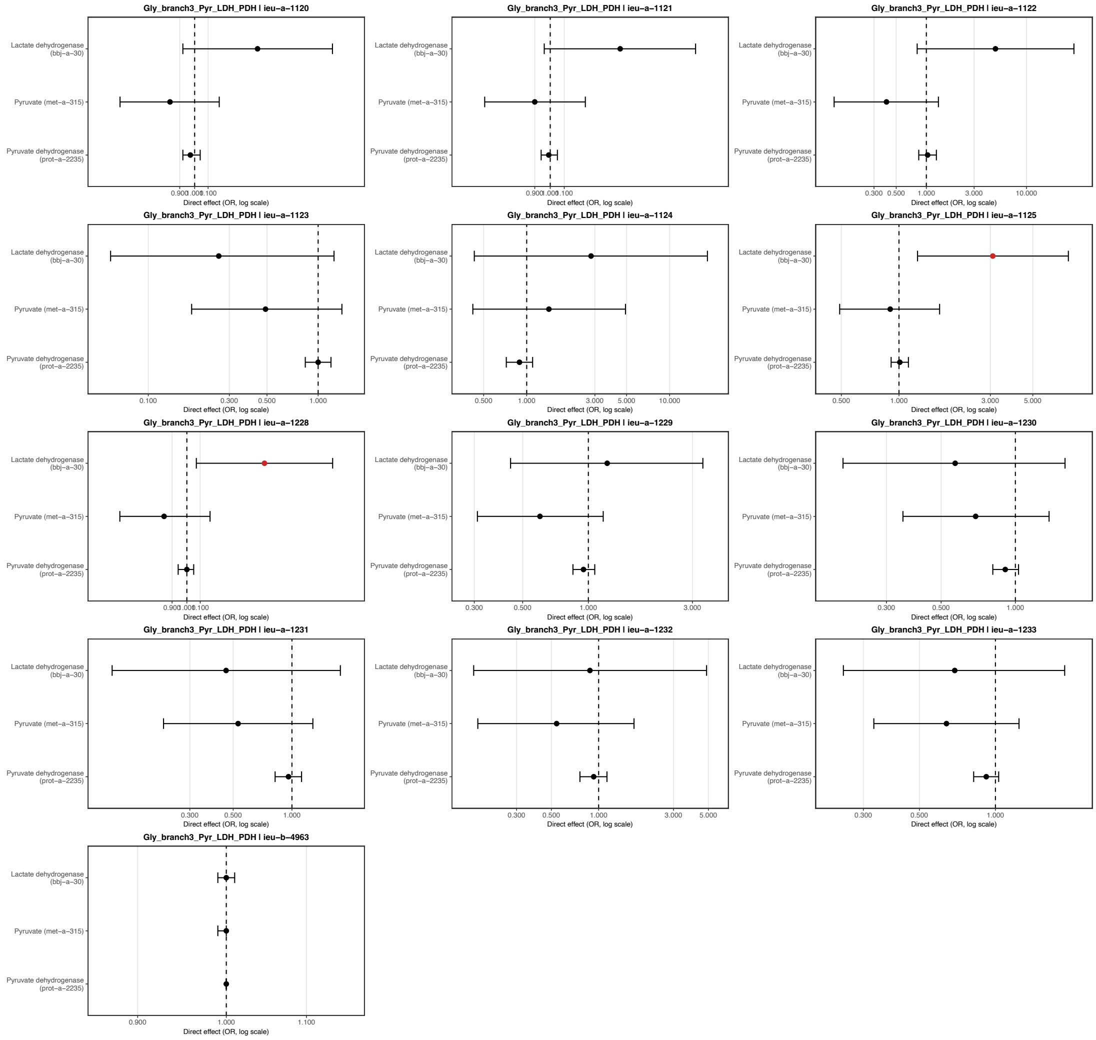

Supplementary Figure S14. Panel D (glycolysis): outcome-wide single-page forest plot for Gly_branch3_Pyr_LDH_PDH.

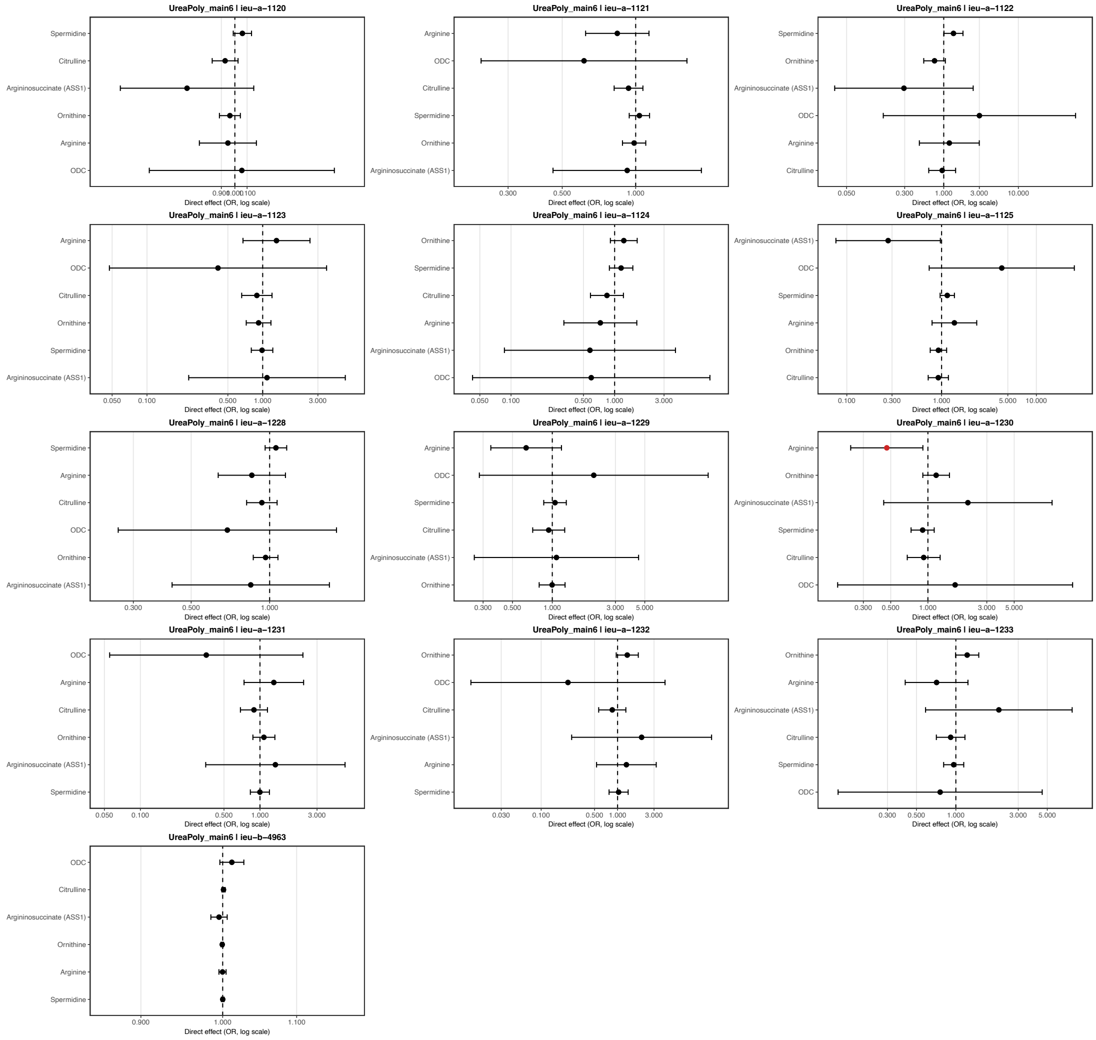

Supplementary Figure S15. Panel E (urea/polyamine): outcome-wide single-page forest plot for UreaPoly_main6.

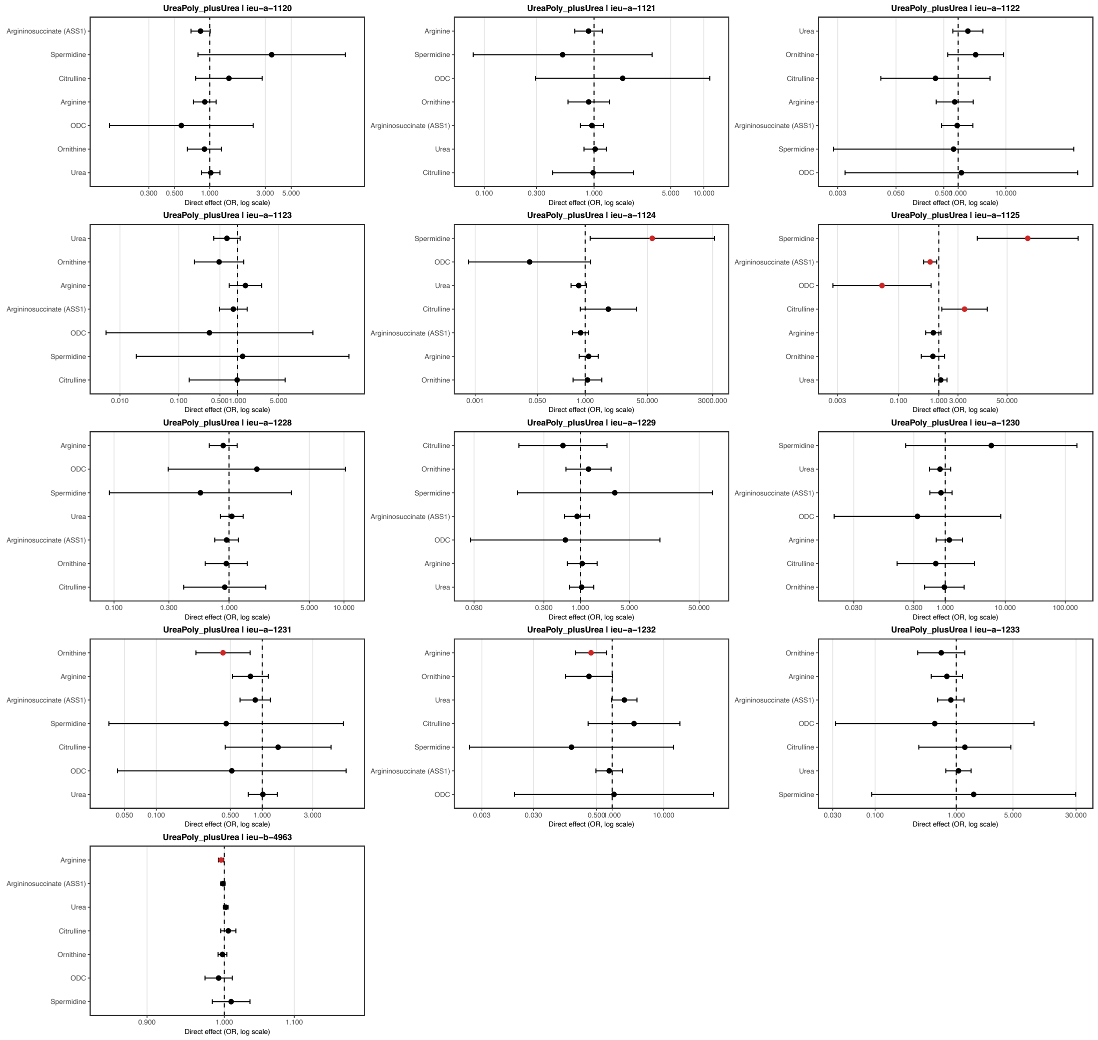

Supplementary Figure S16. Panel E (urea/polyamine): outcome-wide single-page forest plot for UreaPoly_plusUrea.

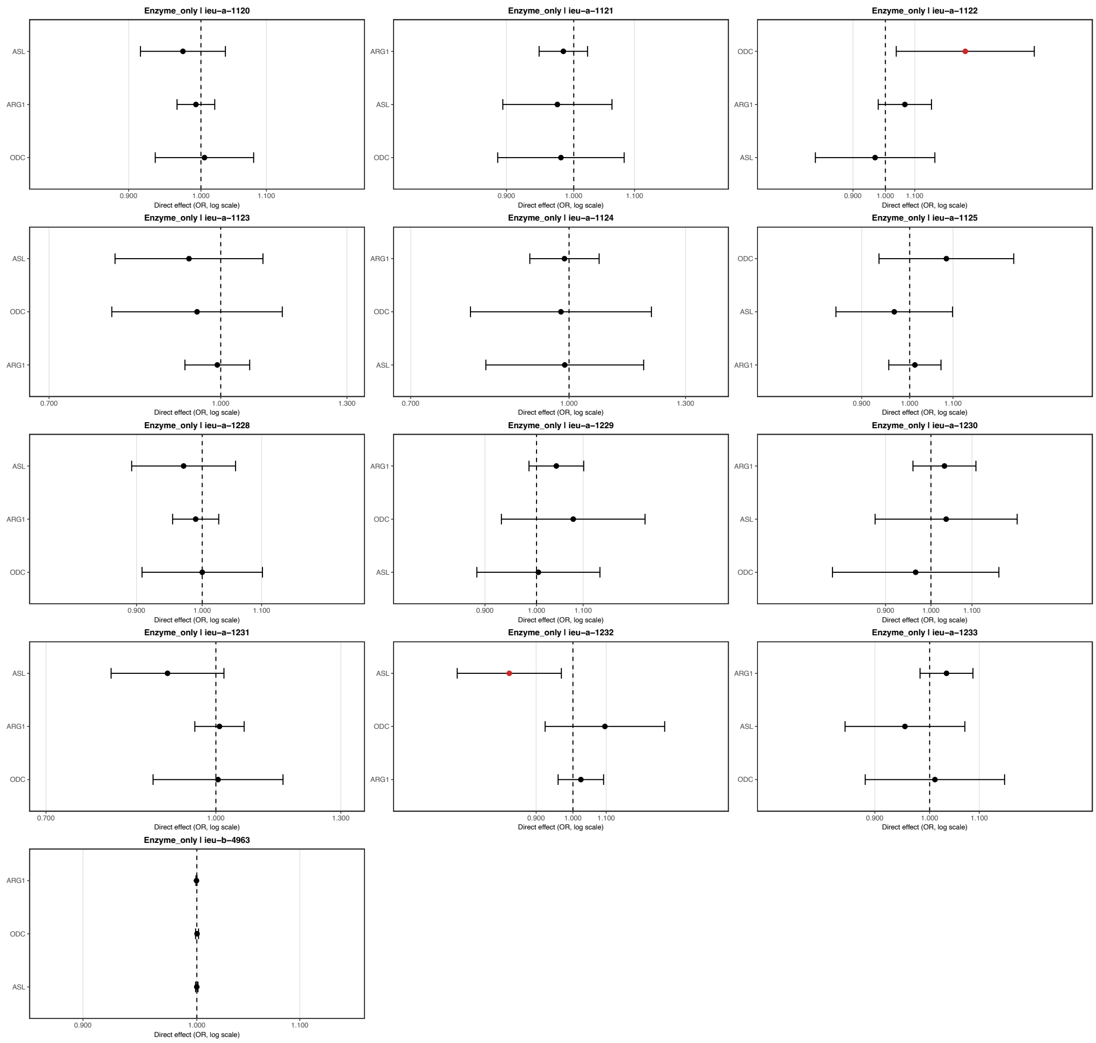

Supplementary Figure S17. Panel E (urea/polyamine): outcome-wide single-page forest plot for Enzyme_only.

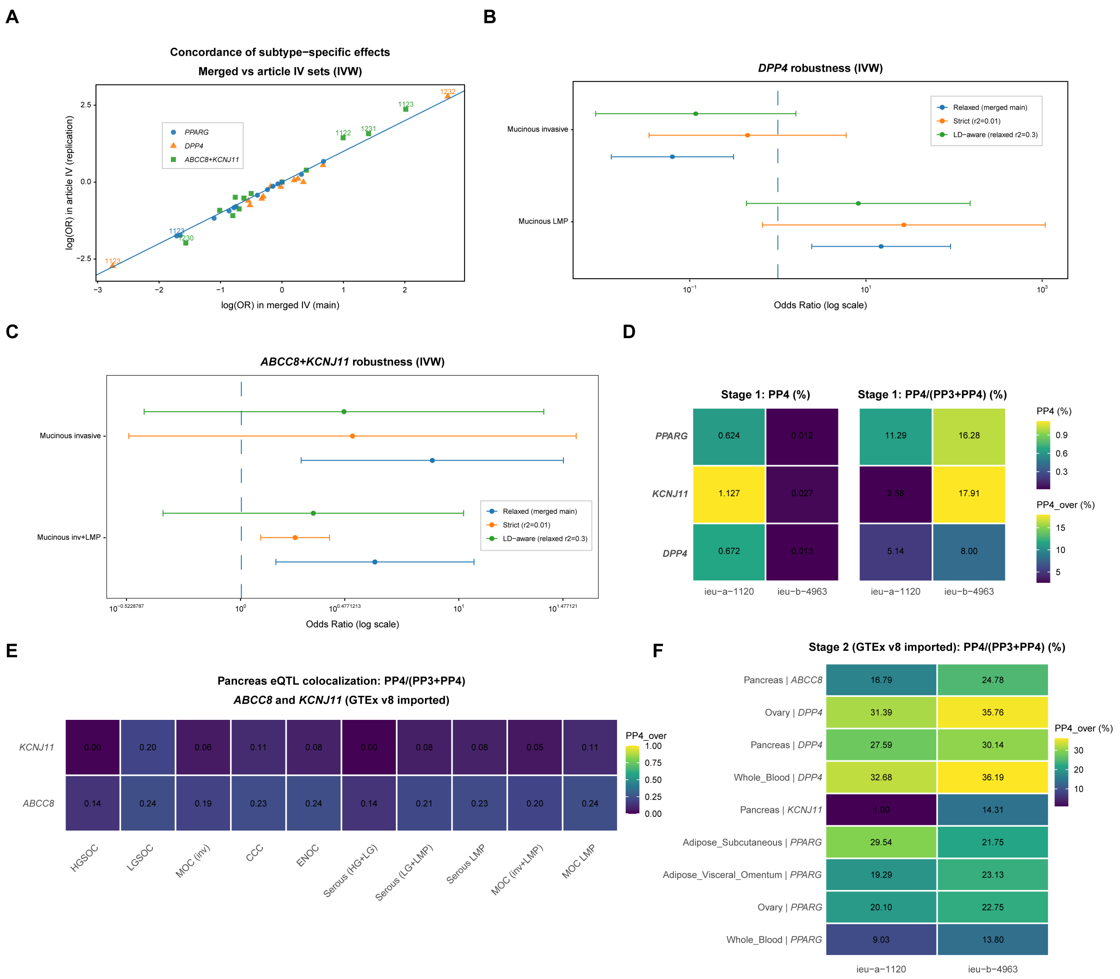

Supplementary Figure S18. Additional robustness analyses for target MR and colocalization prescreening/diagnostics.

(A) Concordance scatter plot for merged versus article instrument sets; **(B–C)** robustness comparisons for *DPP4* and *ABCC8/KCNJ11* under strict clumping and LD-aware settings; **(D–F)** prescreening/diagnostic heatmaps for colocalization (including PP4_over).

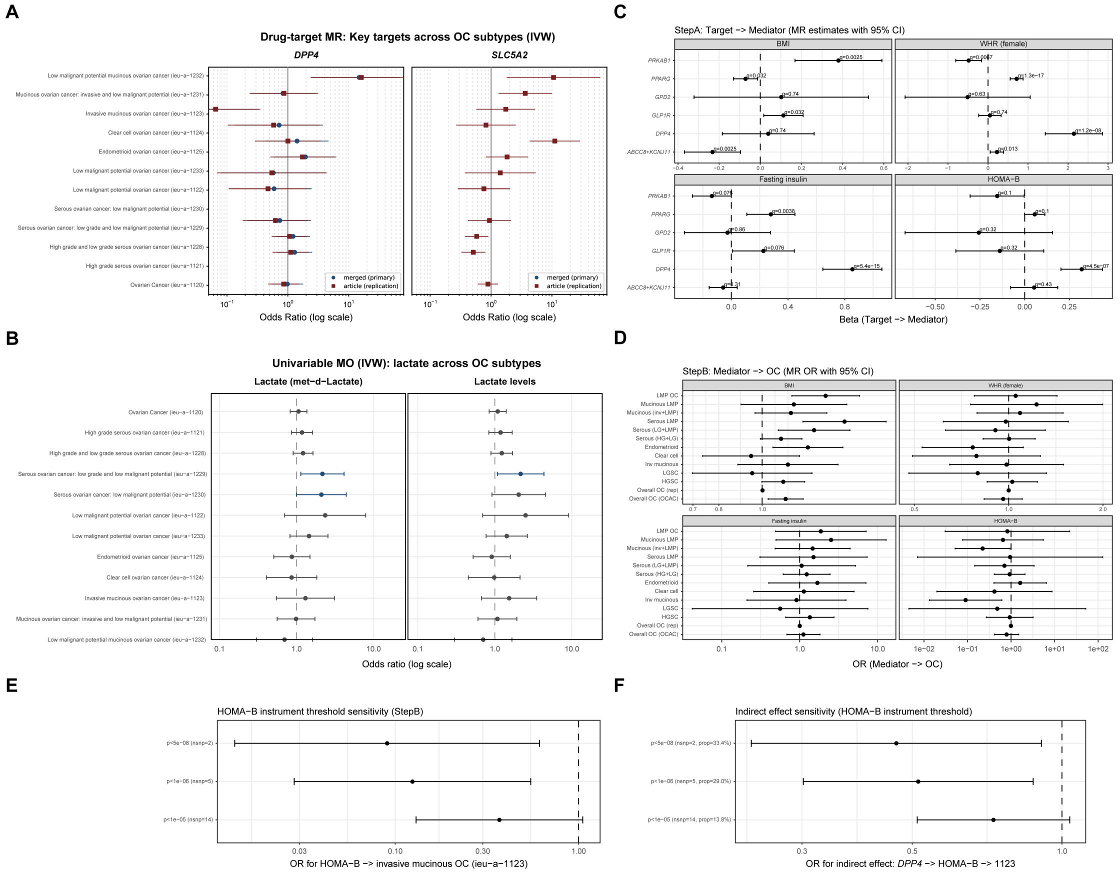

Supplementary Figure S19. Additional analyses for triangular evidence and mediation (including HOMA-B instrument-threshold sensitivity).

(A) Target MR results for additional key targets (e.g., *DPP4, SLC5A2*); **(B)** univariable MR results for lactate traits (lactate and lactate levels) across OC outcomes; **(C–D) c**omponents of two-step MR (target→clinical mediator β; clinical mediator→outcome OR); **(E–F)** Step B and indirect-effect sensitivity analyses across HOMA-B instrument thresholds.

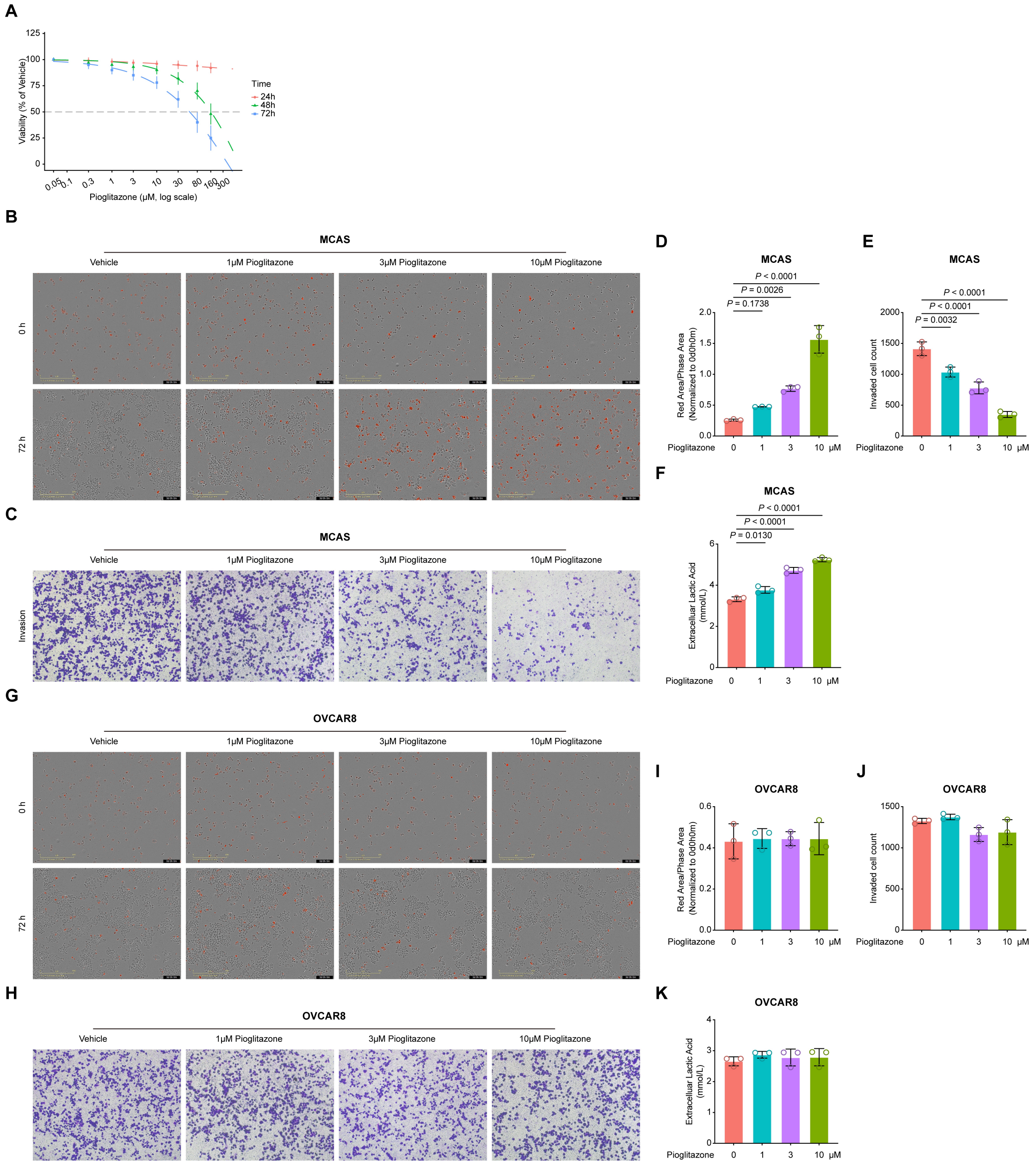

Supplementary Figure S20. Supplementary validation of pioglitazone responses in different ovarian cancer cell lines.

(A) CCK-8 dose–response analysis was performed in RMUG-S cells to evaluate the effect of pioglitazone on cell viability and to define the working conditions for subsequent experiments; 10 µM and 72 h were selected for follow-up studies. **(B–F)** In the mucinous ovarian cancer cell line MCAS, different concentrations of pioglitazone were evaluated for their effects on Incucyte-assessed cytotoxicity (B, with quantification shown in D), Transwell invasion (C, with quantification shown in E), and extracellular lactate levels (F). **(G–K)** In the high-grade serous ovarian cancer cell line OVCAR8, different concentrations of pioglitazone were evaluated for their effects on Incucyte-assessed cytotoxicity (G, with quantification shown in I), Transwell invasion (H, with quantification shown in J), and extracellular lactate levels (K). Data are presented as mean ± SD from n=3 independent experiments. Multiple-group comparisons were analyzed using one-way ANOVA.
