# Supplementary material for: From Pathway Tracing to Actionable Targets: Integrative Mendelian Randomization and Experimental Triangulation Map Metabolic Pathways Across Ovarian Cancer Histotypes
Source: Int J Mol Sci. 2026 Jun 2;27(11):5043. doi: 10.3390/ijms27115043 (PMC13256907; doi:10.3390/ijms27115043)
Supplement: Supplementary file 1 [file ijms-27-05043-s001.zip › Supplementary Table Legends.pdf]

Supplementary Tables

Supplementary Table S1. Full metadata table for GWAS sources and MR design/parameters.

Comprehensive metadata for exposure/mediator/covariate and outcome GWAS (OpenGWAS ID, sample size, ancestry, units/scale), with instrument construction parameters (P threshold, LD clumping), harmonization rules, primary estimation (IVW; Wald ratio for single-SNP instruments), and available sensitivity methods.

Supplementary Table S2. Source information for all 1,400 metabolic traits.

OpenGWAS IDs, sample size, ancestry, platform/matrix, units/scale, and references for all metabolites/ratios.

Supplementary Table S3. Full list of IVW-nominated metabolites for two overall OC outcomes.

Metabolites with IVW $P<0.05$ in ieu-b-4963 and ieu-a-1120, reporting IVW ORs (95% CIs) and P values, with MR-Egger/weighted median results when available.

Supplementary Table S4. Full KEGG pathway analysis results for two overall OC outcomes (MetaboAnalyst).

KEGG enrichment and topology outputs for ieu-b-4963 and ieu-a-1120, including raw P, multiple-testing corrections, pathway impact, and hits/total.

Supplementary Table S5. Full pathway-tracing MR outputs and plotting/mapping tables.

Complete univariable MR results (long/wide formats), q values (if applicable), pathway/outcome mappings, ordering information, and the positive-pair lists used for plotting.

Supplementary Table S6. Full IVW-MVMR results and diagnostics for Panels B–E (four sheets).

Four sheets (amino acids, TCA, glycolysis, urea/polyamine), each including model definitions (exposure composition), model diagnostics (n_union, n_common, n_tests, conditional F, etc.), and full outcome-by-model IVW-MVMR results (including q).

Supplementary Table S7. Drug-target MR results across all targets, outcomes, and instrument sources.

Complete MR results for merged/article/online instrument sets and multiple MR methods (IVW-only and all-methods).

Supplementary Table S8. Colocalization Top20 and merged full results.

Top20 and merged full colocalization outputs (including PP3/PP4), with outcome label mappings.

Supplementary Table S9. Summary of negative/weak colocalization evidence for overall OC.

Transparent summaries for overall OC colocalization results, including PP4/PP4_over and top tissues.

Supplementary Table S10. Sensitivity analyses for drug-target MR using strict independent instruments and LD-aware correlated-SNP models (with diagnostics).

Comparisons of strict and LD-aware settings for key targets, including diagnostic fields.

Supplementary Table S11. Complete two-step MR mediation, MVMR validation, and sensitivity analyses.

Step A, Step B, total effects, indirect effects (with mediation proportion), key-pair summaries, HOMA-B threshold sensitivity, and QC/metadata documentation.
